# Multilayered Cultures of NSCLC cells grown at the Air-Liquid Interface allow the efficacy testing of inhaled anti-cancer drugs

**DOI:** 10.1038/s41598-018-31332-6

**Published:** 2018-08-27

**Authors:** Dania Movia, Despina Bazou, Yuri Volkov, Adriele Prina-Mello

**Affiliations:** 1 Department of Clinical Medicine/Trinity Translational Medicine Institute (TTMI), Trinity College Dublin, Dublin, Ireland; 2 Mater Misericordiae University Hospital, Dublin, Ireland; 3 AMBER Centre, CRANN Institute, Trinity College Dublin, Dublin, Ireland; 4 Department of Histology, Cytology and Embryology, First Moscow State Sechenov Medical University, Moskva, Russian Federation

## Abstract

Evidence supports the advantages of inhalation over other drug-administration routes in the treatment of lung diseases, including cancer. Although data obtained from animal models and conventional *in vitro* cultures are informative, testing the efficacy of inhaled chemotherapeutic agents requires human-relevant preclinical tools. Such tools are currently unavailable. Here, we developed and characterized *in vitro* models for the efficacy testing of inhaled chemotherapeutic agents against non-small-cell lung cancer (NSCLC). These models recapitulated key elements of both the lung epithelium and the tumour tissue, namely the direct contact with the gas phase and the three-dimensional (3D) architecture. Our *in vitro* models were formed by growing, for the first time, human adenocarcinoma (A549) cells as multilayered mono-cultures at the Air-Liquid Interface (ALI). The *in vitro* models were tested for their response to four benchmarking chemotherapeutics, currently in use in clinics, demonstrating an increased resistance to these drugs as compared to sub-confluent monolayered 2D cell cultures. Chemoresistance was comparable to that detected in 3D hypoxic tumour spheroids. Being cultured in ALI conditions, the multilayered monocultures demonstrated to be compatible with testing drugs administered as a liquid aerosol by a clinical nebulizer, offering an advantage over 3D tumour spheroids. In conclusion, we demonstrated that our *in vitro* models provide new human-relevant tools allowing for the efficacy screening of inhaled anti-cancer drugs.

## Introduction

Lung cancer is the leading cause of cancer deaths worldwide^1^. Among other factors, poor prognosis of lung cancer patients is determined by modest or inadequate drugs’ efficacy^2^. The current methods used to administer chemotherapeutics for lung cancer treatment (namely, intravenous injection or oral ingestion) are a constituent component of the problem, causing poor drug responses in human.

Evidence supports the potential advantages of inhalation over intravenous/oral drug administration routes in the treatment of respiratory diseases^3^ such as lung cancer^4^. Despite suffering from poor lung deposition^5^, which may cause inadequate patient compliance, inhalation allows for the administration of lower drug doses than the systemic delivery. This is considered the main advantage of inhalation drug administration. Such advantage derives from the delivery of the active principle directly to the site-of-action and the avoidance of the first-pass metabolism. This offers a faster onset of therapeutic action, and also minimizes the number and severity of systemic adverse effects triggered by the administered drug^6,7^. In addition, inhalation is a needle-free non-invasive administration method, which increases the patients’ acceptance of treatment regimens. The clinical translation of inhaled chemotherapeutics is however impaired by the complete lack of preclinical models capable of predicting the behaviour and action of such compounds in humans. The aim of this study is to facilitate such translation by developing novel *in vitro* models of non-small-cell lung cancer (NSCLC) with increased predictive capability of the efficacy of inhaled anti-cancer agents.

To date, preclinical studies on inhaled compounds have been relying mainly on small animal models (particularly rodents)^8^, which however do not mimic the anatomy of the human respiratory tract^9^. For instance, human lungs have a symmetrical dichotomous branching pattern, whereas rodents have long tapering irregular monopodial airways with small lateral branches. A number of studies have reported that variations in the branching pattern of the airways can lead to differences in the regional deposition of inhaled compounds in the lungs^10^. Importantly, the tracheal length of each animal species also differs: humans have a relatively short trachea compared to other mammals. Similarly, there are apparent differences in the respiration rates. Finally, inhalation pharmacokinetic studies conducted in animals are generally performed using approaches that make the calculation of pharmacokinetic data difficult. For example: liquid intratracheal instillation allows the delivery of a defined dose to the lungs, but often leads to uneven and inhomogeneous lung distribution^11^; the nebulization chamber system allows more precise aerosol delivery to the lungs but it is difficult to accurately determine the dose delivered, as a large proportion of the dose adheres to the rodent’s hair, is then ingested by the animal and contributes to inaccurate pharmacokinetics conclusions.

To overcome the shortfalls of the available *in vivo* models, one could turn to *in vitro* studies. At present, however, *in vitro* alternatives to animal testing for the efficacy assessment of inhaled drugs are unavailable^12^. *In vitro* drug testing relies mainly on the use of cell lines and sub-confluent monolayers (2D)^13^, which are in fact not fully representative of the human tissue architecture, function and signalling. Focusing on *in vitro* systems for cancer research, very few examples of engineered *in vitro* models aiming at incorporating the complexity of the disease pathophysiology) are reported in the scientific literature^14,15^.

The state-of-the-art *in vivo* and *in vitro* lung tumour models presented above highlight the compelling need for the development of preclinical models ensuring that the data generated bears a higher relevance to humans than animal studies or conventional *in vitro* testing based on 2D cultures. This will minimize the technical and physiological gap impacting the translation of inhaled anticancer drugs into clinical practice^16^. Three-dimensional (3D) cultures can satisfy this need. These *in vitro* platforms are in fact better models of the biological and biochemical characteristics of human tissues than conventional 2D cell cultures.

In cancer research, tumour spheroids are the most exploited 3D alternative model to animal studies^17^. However, 3D spheroids do not mimic the direct contact of the lung epithelium with the gas phase, a key feature of the respiratory tract structure and function. Subsequently, these cultures are not suitable models for testing the efficacy of aerosolized drugs.

In the past decades, attempts to simulate the *in vivo* microenvironment of the tumour tissue led researchers to use 3D post-confluent^18,19^ or plateau-phase tumour cell cultures^20–22^. Post-confluent models, also known as Multilayered Cell Cultures (MCCs), are formed by exploiting the ability of some cancer cell lines to grow in multilayers after reaching confluence^23^. Like 3D tumour spheroids, MCCs have been shown to reflect many properties of solid tumours^24^, including drug distribution into the tumour tissue^25,26^. For this reason, these *in vitro* models have been successfully applied for studying the penetration of various chemotherapeutics that are in clinical use^27–33^ or that are still at experimental stage^34–37^ through the tumour tissue. Only a few studies, however, have applied MCCs for studying drug efficacy although, in anticancer drug testing, these systems have shown a degree of selectivity similar to that found in human patients^38–41^. Since MCCs are generally grown on permeable supports^24^, we speculated that they could also be cultured at the Air-Liquid Interface (ALI), in direct contact with the gas phase, to simulate the NSCLC microenvironment. Thus, in our work, we formed, for the first time, ALI MCCs of human adenocarcinoma (A549) cells. By adopting the ALI culturing conditions, our MCCs also enabled the testing of four anti-cancer drugs delivered by a clinical nebulizer in the form of a liquid aerosol.

## Results

### Human adenocarcinoma (A549) cells can be grown as 3D multilayered mono-cultures in ALI conditions

**Figure 1:**
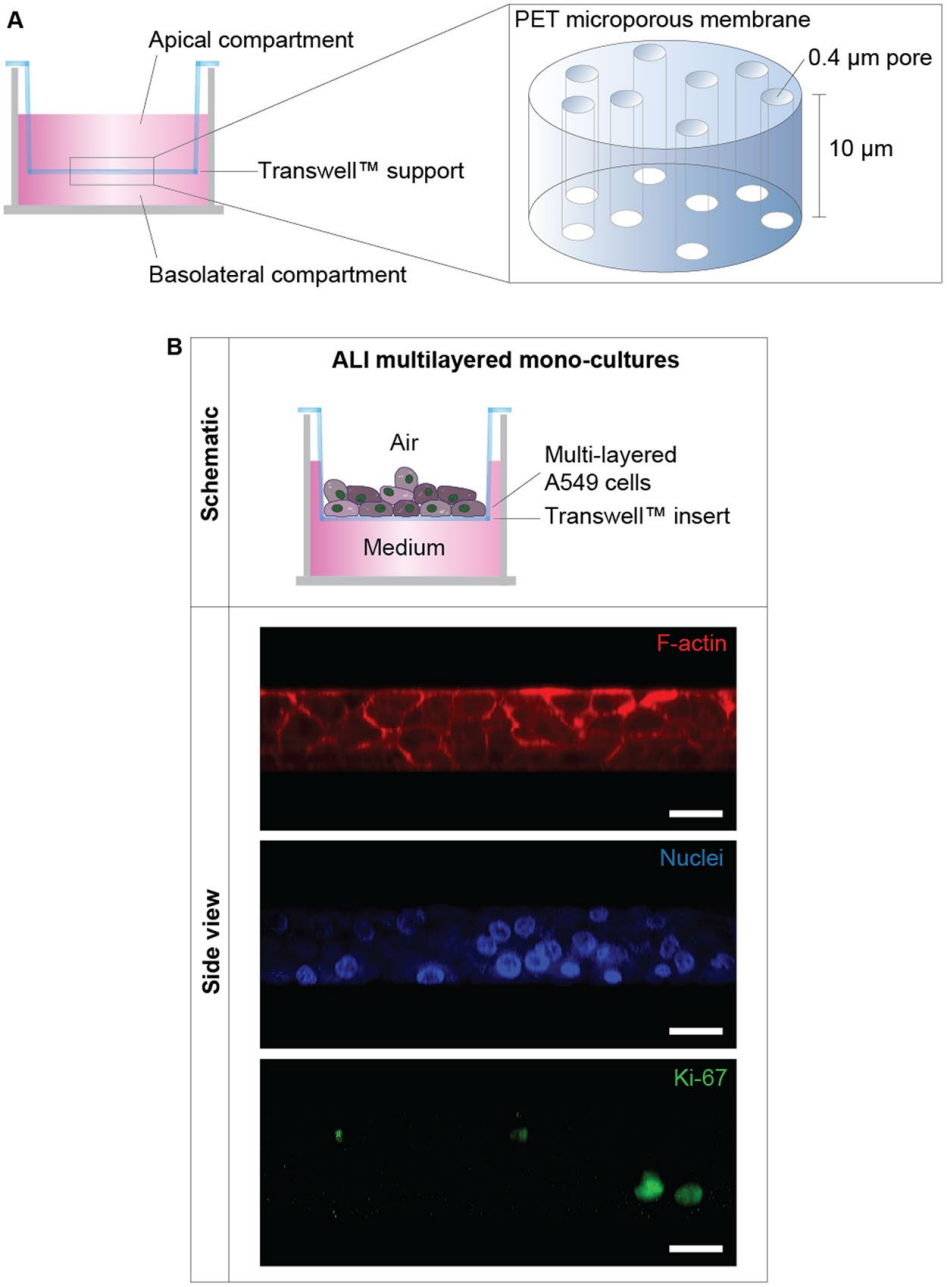
ALI MCCs structure: (**A**) Schematics of the Transwell^TM^ supports used to form the MCCs. (**B**) Schematics of the ALI multilayered mono-cultures developed. Representative LSCM images are also reported, showing the organization of the F-actin (in red) and of cell nuclei (in blue) in these cultures. The Ki67 protein expression throughout the layers is also shown (in green). The Z-stack LSCM images, clearly demonstrating the 3D architecture of the model developed, were reconstructed with ImageJ software to obtain the side view shown. Scale bars: 20 μm (objective lens: 63×).

A549 cells were cultured at high density in ALI conditions on Transwell™ supports (Fig. 1A). These cells successfully formed proliferating 3D multilayers mono-cultures (Fig. 1B). The thickness of the cultures ranged between 3 to 4 layers, as shown by LSCM images (Fig. 1B).

**Figure 2:**
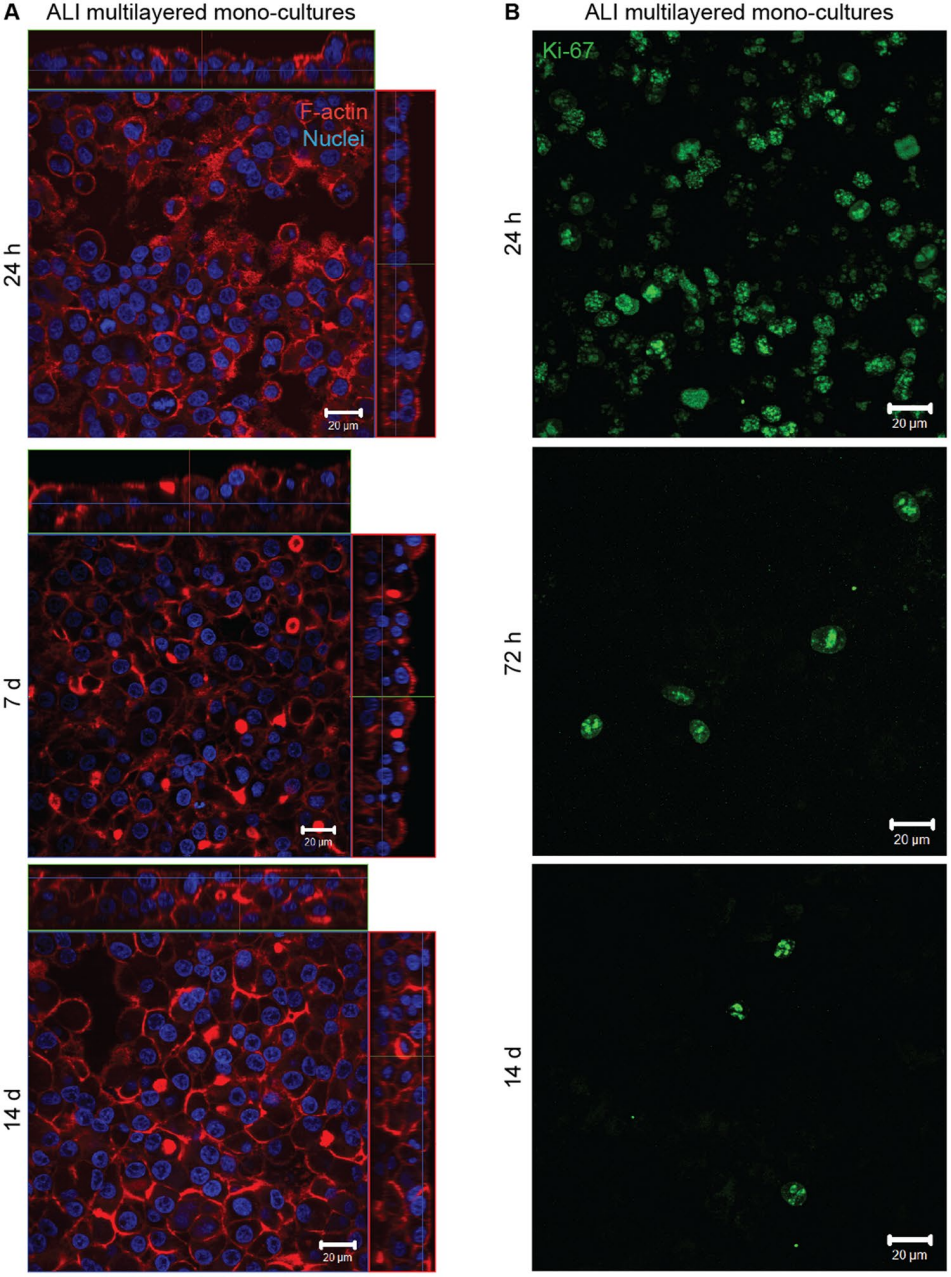
Properties of ALI MCCs: Representative LSCM images of the (**A**) F-actin organization (in red) and (**B**) Ki67 protein expression (in green) in ALI multilayered mono-cultures at different time-points. Full datasets for all time-points are reported in the Supporting Information. Scale bars: 20 μm (objective lens: 63×). (**A**) Cell nuclei were also stained with Hoechst 33342 (in blue). Z-stack images, here presented in orthogonal view, clearly demonstrate the multilayered structure of the *in vitro* models developed. (**B**) Z-stack images of the apical side of the cultures were reconstructed and are shown as three-dimensional projections.

Consistent with the scientific literature^42–46^, our results confirmed that the 3D spatial architecture of the cultures influenced the F-actin organization within the cells. While A549 cells in 2D are well-known to be characterized by F-actin stress-fibers^47^, in ALI multilayered mono-cultures the adenocarcinoma cell line showed a cortical organization of the F-actin (Fig. 2A, and Supporting video VS1). Although the thickness of the cell multilayered models increased incrementally over 14 days of culture, the F-actin organization did not modify overtime (Supporting Figure S1).

Proliferative activity (here quantified as Ki67 protein expression) was detected in A549 cells over 14 d in culture, with Ki67-positive cells found in all layers and observed throughout the cultures (Fig. 2B and Supporting Figure S1). Consistent with previous literature findings^48,49^, in our study, the maximum expression of the Ki67 protein in adenocarcinoma cells was detected at 24 h, and decreased overtime (Fig. 2B and Supporting Figure S1). As expected, Ki67 protein expression was mainly co-localized with the cell nuclei of A549 cells in ALI multilayered mono-cultures, at all time-points tested (Supporting Table S1).

**Figure 3:**
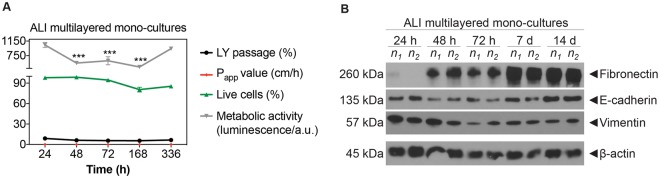
Time-dependent phenotype modifications in ALI MCCs: (**A**) Time-dependent changes in: ATP levels, percentage (%) of live A549 cells, % of LY passage and P_app_ values in ALI multilayered mono-cultures grown up to 14 d. Data are shown as average ± standard error of the mean (n_replicates_ = 2; n_tests_ = 3). The symbols (**) and (***) indicate statistically significant changes as compared to the values measured at 24 h (p < 0.01 and 0.001, respectively) (two-way ANOVA and Bonferroni post-test). (**B**) Western blot analysis of E-cadherin (epithelial marker), vimentin and fibronectin (mesenchymal markers) in A549 cells forming ALI multilayered mono-cultures and cultured up to 14 d. The time-points examined were: 24 h, 48 h, 72 h, 7 d and 14 d. Abbreviations “n_1_” and “n_2_” indicate different experimental replicates. β-actin expression is also reported as proteins loading control.

By quantifying the ATP levels and the percentage of live cells in the cultures, it was found that ALI MCCs models were viable for up to 14 d (Fig. 3A and Supporting Figure S2).

ALI multilayered mono-cultures were not permeable to Lucifer Yellow (LY), a barrier integrity marker, at all time-points, showing P_app_ values and LY passage equal or close to zero (Fig. 3A). This demonstrated that an epithelial barrier was formed at 24 h and that it remained intact over 14 d in culture in both ALI multilayered mono- and co-cultures. The multilayered architecture of the *in vitro* ALI MCCs was sufficient to guarantee a reduced permeability without conferring any measurable Trans-Epithelial Electrical Resistance (TEER) (Supporting Figure S3), one of the key features defining the formation of an epithelial barrier. This is consistent with previous reports on *in vitro* epithelial models formed by A549 cells^50^. This was due to the inability of A549 cells to form functional tight-junctions, as extensively reported in the literature^51,52^.

### ALI multilayered mono-cultures modify their phenotype overtime

Loss of E-cadherin (epithelial marker) is one of the best indicators of EMT transition in epithelial cells^53^. Our results showed that E-cadherin was expressed in ALI multilayered mono-cultures, at all time-points tested (Fig. 3B). Not surprisingly, the mesenchymal protein vimentin was also expressed in cell cultures, at all time-points. Vimentin is, in fact, a protein expressed in lung epithelial cells of mesenchymal origin^54,55^, such as the A549 cell line. Fibronectin expression (a mesenchymal marker) was absent at 24 h but was detected in MCCs after 48 h growth. Thus, we concluded that, overtime, A549 cells acquired metastatic-like properties when grown as ALI MCCs. Please note that the original (full length and uncropped) Western blots are included in a Supplementary Information file (Supporting Figure S4).

### Multilayered architecture confers chemoresistance

**Figure 4:**
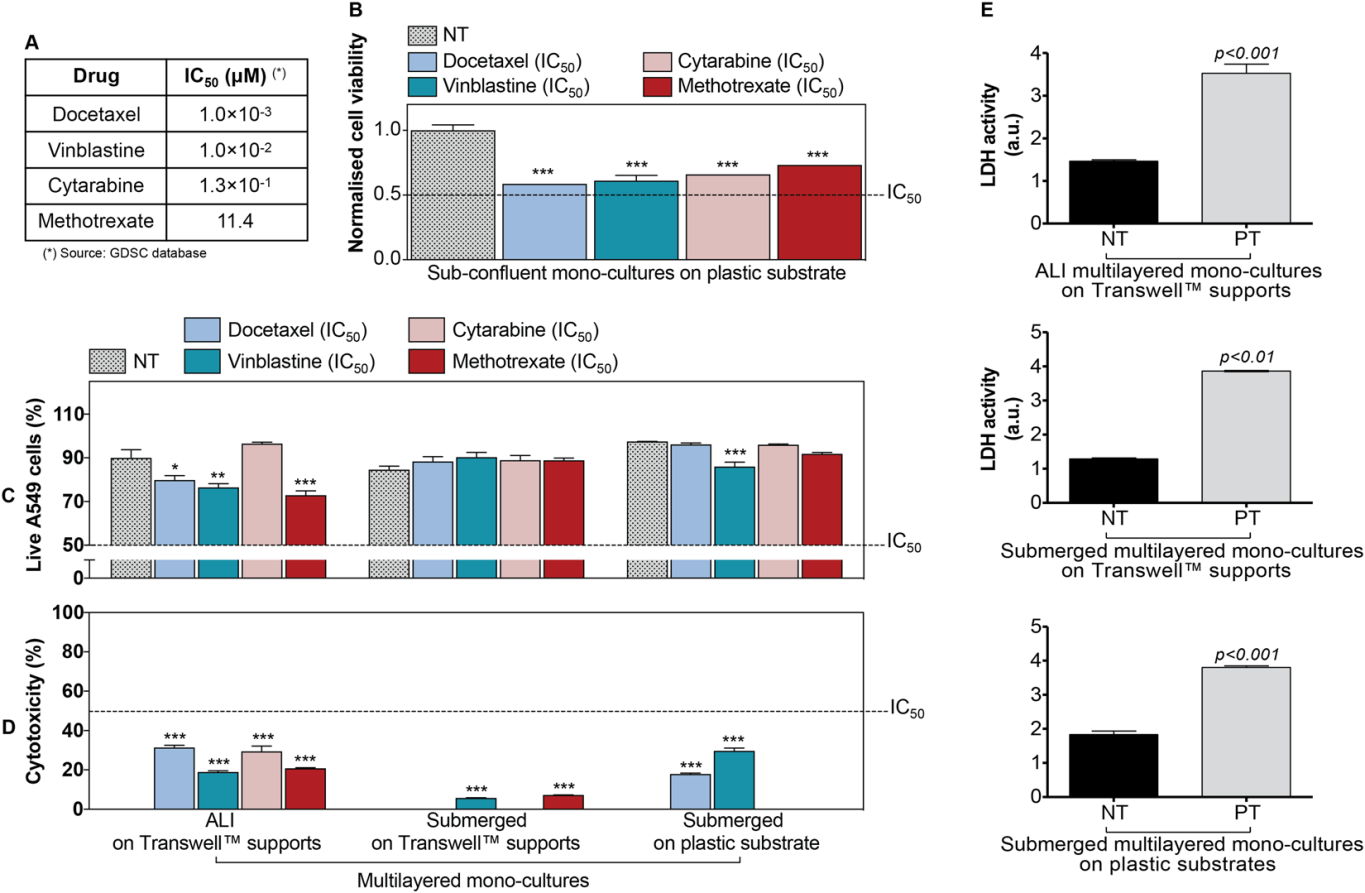
Cell culture architecture influences the response to anti-cancer drugs administered by direct inoculation: (**A**) Details of the four anti-cancer drugs tested in this study. Their half-maximal inhibitory concentration (IC_50_) is listed as reported in the GDSC database for the A549 cell model (in the text referred to as “nominal IC_50_”). (**B**) Changes in cell viability of sub-confluent mono-cultures of A549 cells grown on plastic substrates and exposed to the four anti-cancer drugs at their nominal IC_50_ concentration for 72 h. GDSC database experimental conditions were reproduced in our assay. Data, shown as average ± standard error of the mean (n_replicates_ = 3; n_tests_ = 3), are normalized to the cell viability of the untreated control (NT). The symbol (***) indicates statistically significant differences from NT (p < 0.001) (one-way ANOVA followed by Dunnett post-test). (**C,D**) Percentage (%) of live A549 cells (**C**) and % cytotoxicity (**D**) detected in MCCs cultured for 14 d (from left to right) either on Transwell^TM^ supports (in ALI or in submerged conditions) or on plastic substrates (in submerged conditions) and then exposed to four anti-cancer drugs at their nominal IC_50_ concentration for 72 h. Data are reported as average ± standard error of the mean (n_replicates_ = 2; n_tests_ = 3). The symbols (*), (**) and (***) represent significant differences from the corresponding NT (p values < 0.05, 0.01 and 0.001, respectively) (two-way ANOVA and Bonferroni post-test). (**E**) Histograms of the LDH activity in the experimental controls: untreated mono-cultures (NT) and positive controls (PT). A significant LDH activity was detected in supernatants harvested from PT. Data are reported as average ± standard error of the mean (n_replicates_ = 3; n_tests_ = 3). p < 0.01 and p < 0.001 indicate a significant difference from NT (t-test).

The drug sensitivity of the A549 cell line batch used in this study was validated against the GDSC database benchmark^56^, which was originally derived from dose-response curves. For this validation, sub-confluent monolayered mono-cultures of A549 cells were exposed to four anti-cancer drugs, namely docetaxel, vinblastine, cytarabine and methotrexate. These are in clinical use and they induce different cell death in the cell model tested, with docetaxel being the most effective drug and methotrexate the least. The four chemotherapeutics were tested at their nominal IC_50_ concentration, as reported by the GDSC database for A549 cells (Fig. 4A). Cell line sensitivity was measured following 72 h of drug treatment (Fig. 4B). As predicted during the design of the experiment, a significant decrease (*p* < 0.001) in cell viability was detected in sub-confluent monolayered mono-cultures following exposure to any of the four drugs. Consistently with the data provided by GDSC database, such decrease was equal or close to 50% for all drugs.

Conversely to the above, no cellular response was detected at the drugs’ nominal IC_50_ concentration when A549 cells were cultured as MCCs on plastic substrates or on Transwell^TM^ supports in submerged or ALI conditions (Fig. 4C). For all MCCs, the A549 cells viability remained above 50% following drug treatment (Fig. 4C). Similar results were seen for cytotoxicity, for which the levels always remained below the 50% after 72 h drug exposure (Fig. 4D). As expected, in the positive controls a significant LDH activity was detected (Fig. 4E). Thus, our results proved that multilayered mono-cultures were more chemoresistant than the sub-confluent cell model formed by the same cell line.

Whereas substrates had no influence on MCCs response, our experiments showed that the MCCs chemoresistance was affected by the direct contact with the gas phase (Fig. 4C,D and Supporting Figure S5). Compared to submerged MCCs grown on Transwell^TM^ supports, ALI multilayered mono-cultures showed a lower chemoresistance, evidenced by a significant decrease in the % of live A549 cells and a significant increase in cytotoxicity following drug exposure (Supporting Table S2).

**Figure 5:**
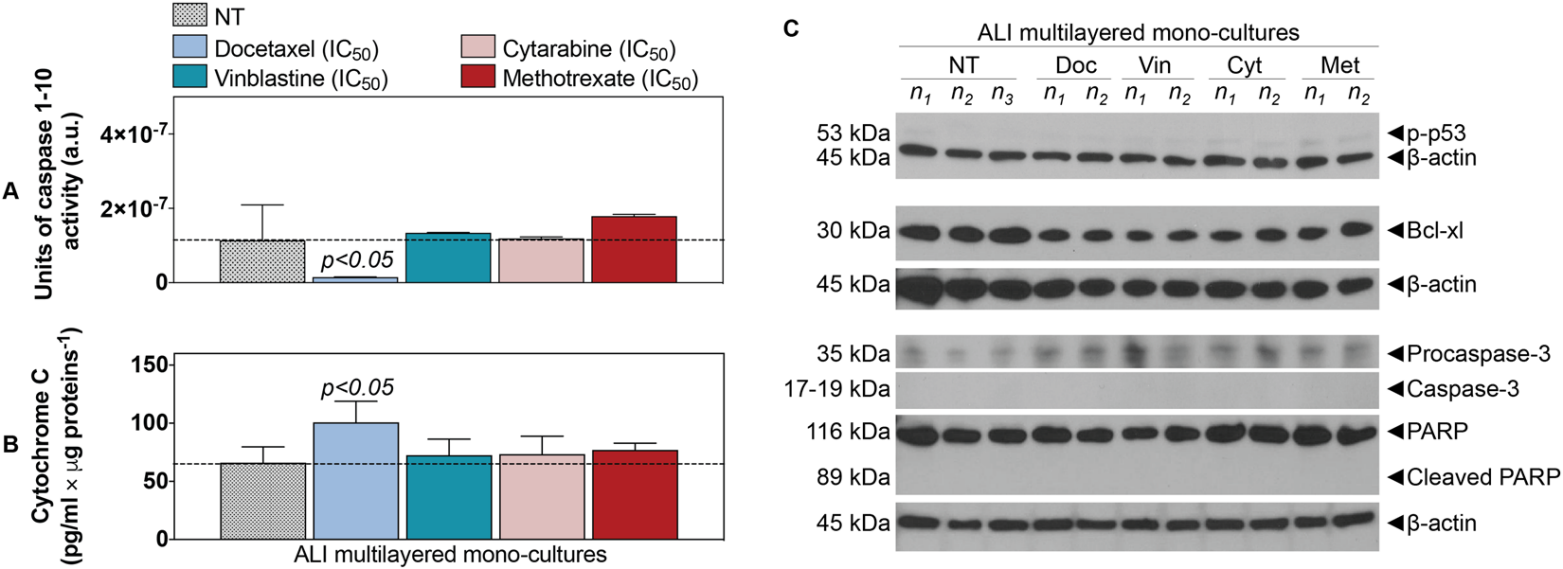
ALI multilayered mono-cultures show chemoresistance: (**A,B**) Histograms showing the (**A**) units of caspases 1–10 activity and (**B**) the levels of cytochrome c released from the mitochondria into the cell cytoplasm, as detected in ALI multilayered mono-cultures grown for 14 d and then exposed to the four anti-cancer drugs at their nominal IC_50_ for 72 h. Cell cultures were exposed to drugs by direct inoculation. Untreated cultures were also tested as negative control (NT). Dotted lines indicate the levels of caspases activity and cytochrome c release by NT. Data are presented as average ± standard error of the mean (n_replicates_ = 2; n_tests_ = 3). p < 0.05 indicates significant differences from NT (one-way ANOVA with Dunnett post-test). (**C**) Western blot analysis of the expression of phospho-p53 (p-p53), Bcl-xl, procaspase-3 and caspase-3, PARP and its cleaved form (cleaved PARP) in A549 cells cultured as ALI multilayered mono-cultures for 14 d and then exposed to docetaxel (Doc), vinblastine (Vin), cytarabine (Cyt) or methotrexate (Met) at their nominal IC_50_ for 72 h. Cell cultures were exposed to drugs by direct inoculation. Untreated cultures (NT) were also analysed. Abbreviations “n_1_”, “n_2_” and “n_3_” indicate different biological replicates. β-actin expression is also reported as proteins’ loading control.

To exclude artefacts associated with the assays adopted that may have affected our chemoresistance results, we investigated the biochemical responses triggered by drug exposure in ALI multilayered mono-cultures. No caspases 1–10 activity could be detected (Fig. 5A). It should be noted that basal levels of caspase activity were detected in untreated cultures (NT) (Fig. 5A). This basal activity of caspases was possibly associated with some housekeeping functions in the cells, as well as involved in caspase-dependent non-lethal cellular processes^57^. Similarly, no significant release of cytochrome c was found when ALI multilayered mono-cultures were exposed to vinblastine, cytarabine and methotrexate, as compared to untreated cultures (NT) (Fig. 5B). Conversely, significant release of cytochrome c could be detected under docetaxel treatment (Fig. 5B). However, no activation of procaspase-3 (into its cleaved form) was evidenced in ALI multilayered mono-cultures exposed to docetaxel (Fig. 5C). Expression of the cleaved, active form of caspase-3 was lacking also in ALI MCCs exposed to vinblastine, cytarabine and methotrexate (Fig. 5C). Our Western blot analysis showed that the lack of procaspase-3 cleavage was reflected by the absence of PARP activation, for all drug treatments (Fig. 5C). Phosphorylation of the p53 protein could also not be detected upon exposure to any of the four drugs (Fig. 5C). Finally, we found that the protein B-cell lymphoma-extra large (Bcl-xl) was expressed in untreated ALI multilayered cultures and was unaffected by drug exposure (Fig. 5C). Please note that the original (full length and uncropped) blots of Fig. 5C are included in a Supplementary Information file (Supporting Figure S6).

### ALI multilayered cultures can reproduce the low efficacy of anticancer drugs administered by aerosol

**Figure 6:**
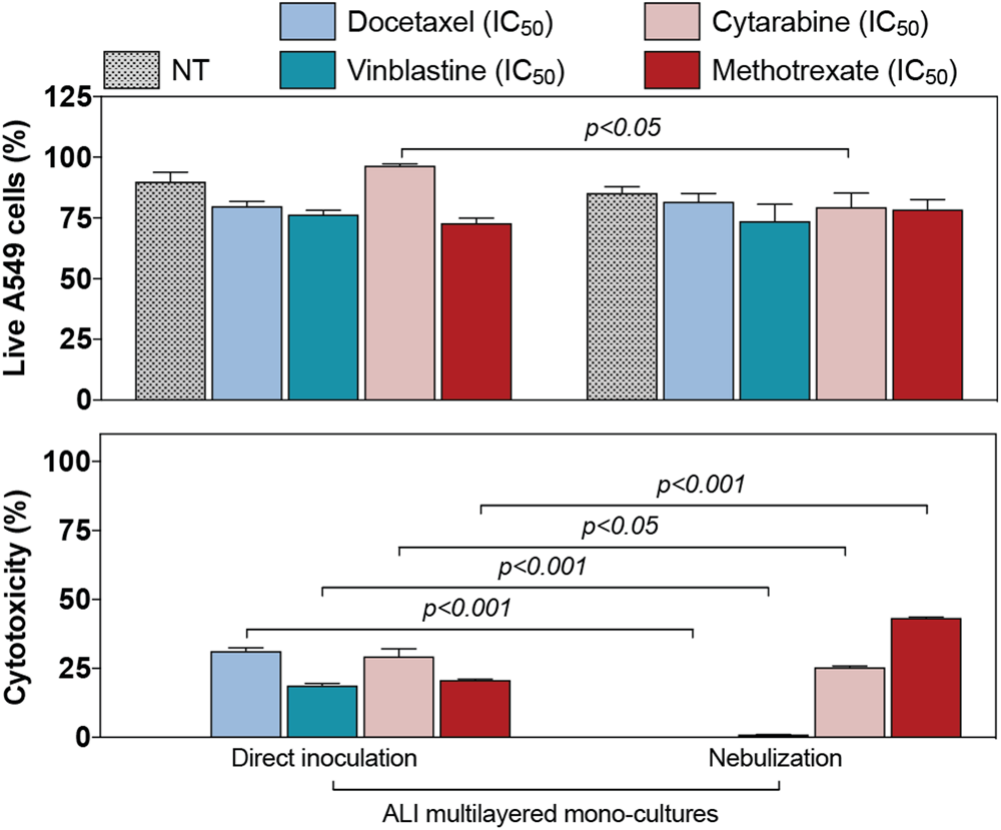
Efficacy of anti-cancer drugs delivered as a liquid aerosol by nebulization in ALI MCCs. Percentage (%) of live A549 cells (top histogram) and cytotoxicity (bottom histogram) detected in ALI multilayered mono-cultures. *In vitro* models were exposed to four anti-cancer drugs (docetaxel, cytarabine, vinblastine and methotrexate) at their nominal IC_50_ concentration for 72 h, by direct inoculation (on the left) or nebulization (on the right). Data are reported as average ± standard error of the mean (n_replicates_ = 2; n_tests_ = 3). p values indicate significant differences (two-way ANOVA and Bonferroni post-test).

As compared to direct inoculation, the cytotoxic effect of anti-cancer drugs (docetaxel, vinblastine, cytarabine and methotrexate) significantly decreased when drugs were administered to ALI multilayered mono-cultures as a liquid aerosol by nebulization (Fig. 6). In particular, a lower cytotoxicity could be detected when the four drugs were nebulized. It should be highlighted here that aerosol-to-cell delivery by the Aeroneb® Pro nebulizer was proved to be reproducible and comparable to the administration by direct inoculation (Supporting Figure S7).

### Chemoresistance of ALI multilayered mono-cultures is comparable to that detected in hypoxic 3D tumour spheroids

**Figure 7:**
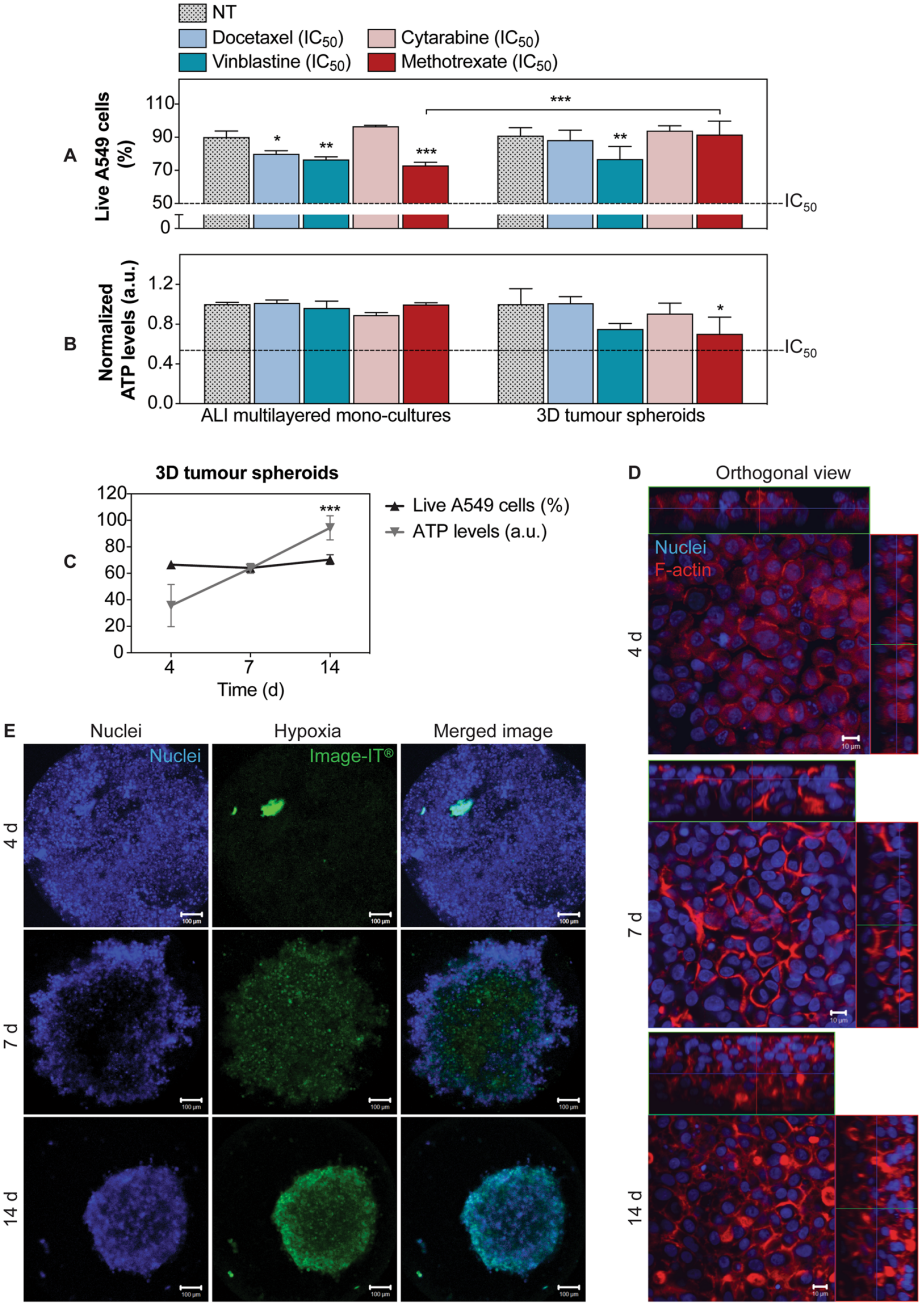
Comparison of the chemoresistance detected in ALI multilayered mono-cultures and 3D tumour spheroids: (**A,B**) Percentage (%) of live A549 cells (**A**) and ATP levels (**B**) detected in (from left to right) ALI multilayered mono-cultures and 3D tumour spheroids. Both *in vitro* models were grown for 14 d and then exposed to four anti-cancer drugs (docetaxel, cytarabine, vinblastine and methotrexate) at their nominal IC_50_ concentration for 72 h. Cell cultures were exposed to drugs by direct inoculation. Data are reported as average ± standard error of the mean (n_replicates_ = 2; n_tests_ = 3). The symbols (*), (**) and (***) indicate significant differences (p values < 0.05, 0.01 and 0.001, respectively) (two-way ANOVA and Bonferroni post-test). (**C**) Changes in cell viability in 3D tumour spheroids cultured for 4, 7 and 14 d and analysed for their percentage of live cells (in black) and total ATP levels (in grey). Data are shown as average ± standard error of the mean (n_replicates_ = 2; n_tests_ = 3). The symbol (***) indicates a significant difference from values at t = 4 d (one-way ANOVA and Dunnett post-test). (**D**) Representative LSCM images of the F-actin organization (in red) in 3D tumour spheroids at different time-points. Cell nuclei were also stained with Hoechst 33342 (in blue). Z-stack images, here presented in orthogonal view, clearly demonstrate the growing thickness of the spheroids overtime. Scale bars: 10 μm (objective lens: 63×). (**E**) Live 3D tumour spheroids stained for cell nuclei (in blue) and hypoxia (in green) at different time-points. Z-stack images of the cultures were reconstructed and are shown as three-dimensional projections. Scale bars: 100 μm (objective lens: 10×).

ALI multilayered mono-cultures and 3D tumour spheroids were exposed to the chosen anti-cancer drugs docetaxel, vinblastine, cytarabine and methotrexate at their nominal IC_50_ for 72 h. Their viability was then evaluated as % of live A549 cells (Fig. 7A) and total ATP levels (Fig. 7B). Our results clearly showed that, in both cell models, cell viability remained well above the 50% threshold for all drugs. This indicated that both models were chemoresistant. In addition, chemoresistance was generally comparable between the two cultures (Fig. 7A,B).

Similar to ALI multilayered mono-cultures, the 3D tumour spheroids tested herein were grown for 14 d. At this time point, the spheroids shared many features of the ALI MCCs including high cell viability (Fig. 7C and Supporting Figure S8), a thick multilayered structure with cortical F-actin organization (Fig. 7D and Supporting Figure S9) and a mesenchymal phenotype with increased fibronectin expression (Supporting Figure S10). E-cadherin (epithelial marker) was, however, completely down-regulated in 3D tumour spheroids (Supporting Figure S10), while it was still present in ALI multilayered mono-cultures (Fig. 3). This is consistent with the literature showing that A549 cells spheroids can display a loss of E-cadherin expression and elevated levels of mesenchymal markers, such as fibronectin^58^. As expected^59^, the 3D tumour spheroids were characterized by hypoxic regions (Fig. 7E).

### Overexpression of MRP1/ABCC1 drug efflux pump drives ALI multilayered mono-cultures multidrug resistance (MDR)

**Figure 8:**
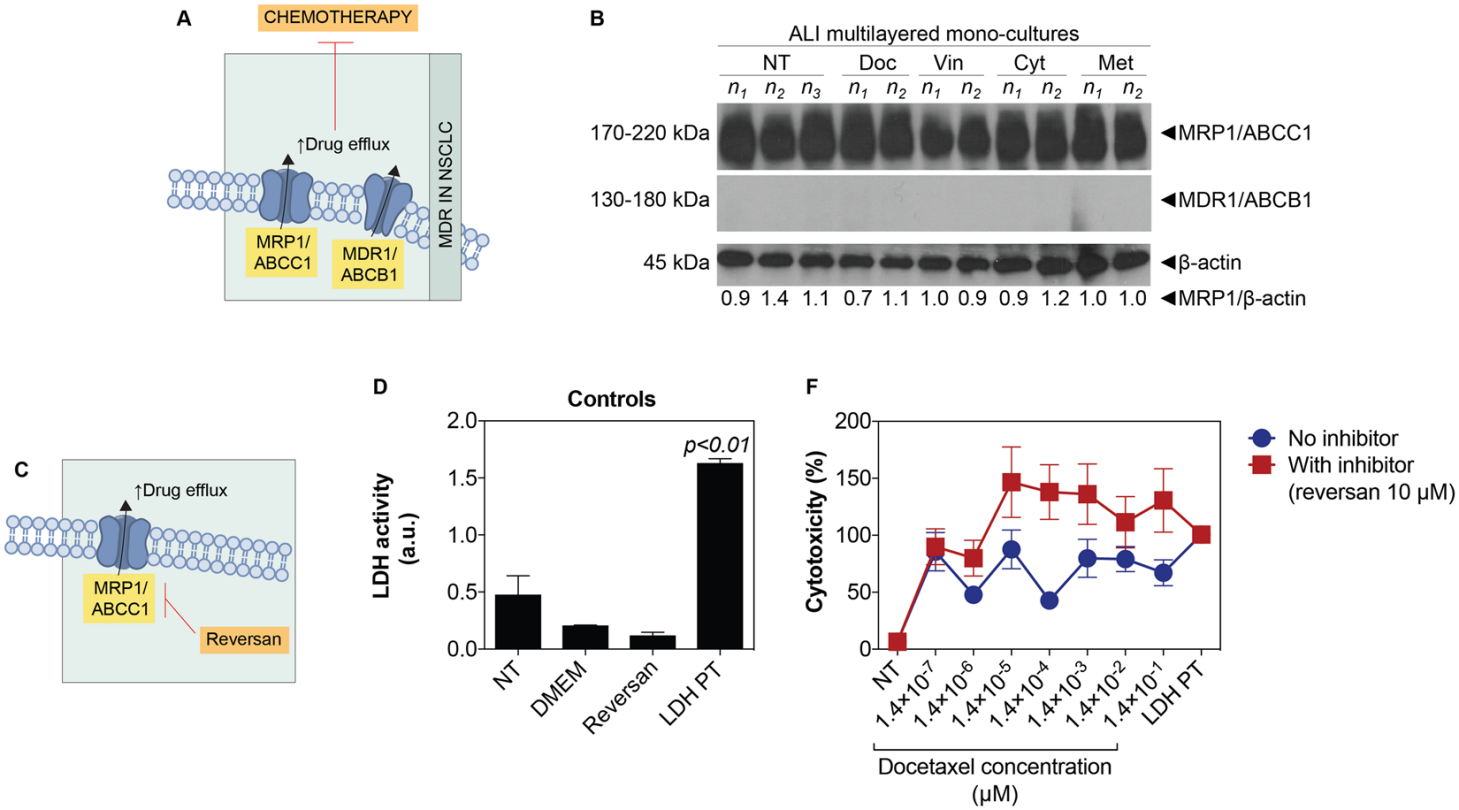
MDR mechanism in ALI multilayered mono-cultures: (**A**) Schematics of MDR in cancer cells triggered by overexpression of MRP1/ABCC1 and MDR1/ABCB1 drug efflux pumps. (**B**) Western blot analysis of the expression of MRP1/ABCC1 and MDR1/ABCB1 drug efflux pumps in A549 cells forming ALI multilayered mono-cultures grown for 14 d and then exposed to docetaxel (Doc), vinblastine (Vin), cytarabine (Cyt) or methotrexate (Met) at their nominal IC_50_ concentration for 72 h. Cell cultures were exposed to drugs by direct inoculation. The expression of these pumps in untreated cultures (NT) is also reported for comparison. Abbreviations “n_1_”, “n_2_” and “n_3_” indicate different experimental replicates. β-actin expression is reported as proteins loading control. (**C**) Schematics of the mechanism of action of reversan, a selective MRP1/ABCC1 inhibitor. (**D**) Histogram of the LDH activity in the experimental controls: untreated ALI multilayered mono-cultures (NT), cell-free supplemented cell medium (DMEM), ALI multilayered mono-cultures exposed to reversan (10 μM) for 72 h, and positive control (LDH PT). No significant LDH activity was detected following reversan treatment. Data are reported as average ± standard error of the mean (n_replicates_ = 2; n_tests_ = 3). p < 0.01 indicates a significant difference from NT (one-way ANOVA with Dunnett post-test). (**F**) Percentage (%) cytotoxicity detected by LDH cytotoxicity assay in ALI multilayered mono-cultures grown for 14 d and exposed to 10 concentrations of docetaxel for 72 h, in the presence or absence of reversan (10 μM). Cell cultures were exposed to drugs by direct inoculation. Values for untreated cultures (NT) and positive control (LDH PT) are also shown. Data are reported as average ± standard error of the mean (n_replicates_ = 2; n_tests_ = 3). Differences were not significant (two-way ANOVA with Bonferroni post-test).

One of the most important mechanisms underlying MDR in NSCLC is overexpression of adenosine triphosphate (ATP)-binding cassette (ABC) transporters, such as multidrug resistance proteins (MRPs) and multidrug transporters (MDRs) (Fig. 8A). In our study, untreated ALI multilayered mono-cultures (NT) expressed the drug efflux pump MRP1/ABCC1 but not the MDR1/ABCB1 transporter (Fig. 8B). Of interest, MRP1/ABCC1 expression levels did not change when the *in vitro* cultures were exposed to the four anti-cancer drugs (docetaxel, vinblastine, cytarabine and methotrexate) (Fig. 8B). Please note that the original (full length and uncropped) blots of Fig. 8B are included in a Supplementary Information file (Supporting Figure S11).

MRP1/ABCC1 is known to confer resistance to *Vinca* alkaloids (vinblastine)^60^ and toxic folate analogues, such as methotrexate^61^. In contrast, efflux of chemotherapeutic agents such as taxanes (docetaxel) and nucleoside drugs (cytarabine) is generally driven by the MDR1/ABCB1 transporter^62,63^. This brought us to further investigate whether MRP1/ABCC1 up-regulation in ALI multilayered mono-cultures induced chemoresistance even for those drugs that are generally not associated with MRP1/ABCC1-triggered MDR. To test this hypothesis, we exposed mono-cultures to ten increasing concentrations of docetaxel in the presence of reversan, which is a selective inhibitor of MRP1/ABCC1 and is capable of increasing the sensitivity of MRP1-overexpressing cancer cells to chemotherapy^64^ (Fig. 8C). Reversan was found non-toxic in ALI multilayered mono-cultures (Fig. 8D). Nevertheless, evidence of the increase in docetaxel efficacy was detected when the anti-cancer drug was administered in conjunction with reversan (Fig. 8F).

## Discussion

For the efficacy testing of inhaled anti-cancer drugs, it is important to mimic the direct contact of the lung epithelium with the gas phase. ALI cultures are the only *in vitro* model available that can reproduce this feature. In addition, these *in vitro* models allow aerosols to directly deposit onto semi-dry apical cell surfaces. Also, in ALI *in vitro* models, drug deposition and dissolution occur in a small volume of cell lining fluid and mimic closely the delivery of liquid drug aerosol on the lung surface of human patients. These properties make ALI *in vitro* models ideal candidates for testing inhaled drugs. Based on this knowledge, our study aimed at creating novel *in vitro* models that incorporated both the ALI culturing conditions and the 3D architecture of the tumour tissue, which is generally mimicked in *in vitro* cancer research experiments through the adoption of 3D tumour spheroids. To achieve this, we developed ALI multilayered mono-cultures of A549 cells, and we investigated how the features of such *in vitro* models would affect the cellular response to four different benchmark anti-cancer drugs delivered by direct inoculation or as a liquid aerosol by means of a clinical nebulizer.

During ALI cultures formation, A549 cells overcame contact-mediated inhibition and contributed to the formation of the multilayers (Fig. 1). Scientific literature reports that A549 cells are non-homogeneous and exist in several subpopulations^65–68^. Four main A549 subpopulations can be described based on their morphological and functional characteristics^65,66^. Three of these subpopulations form well-spread monolayers and exhibit contact-mediated growth inhibition. In contrast, A549 cells of a fourth subpopulation can overcome contact-mediated inhibition and exhibit anchorage-independent growth on top of the monolayer formed. We hypothesize that the cells of this subpopulation, which have been demonstrated to be tumorigenic in nude mice^66^, were responsible for the formation of our MCCs. Further studies would be needed to confirm this. Notably, ALI multilayered mono-cultures up-regulated the expression of fibronectin overtime (Fig. 3). It is known that fibronectin stimulates the proliferation of NSCLC cells *in vitro*, through α5β1 integrin receptor-mediated signals^69^. We could hypothesize, therefore, that fibronectin up-regulation was directly involved in promoting the formation of multilayers in our MCCs. Evidence also suggests that fibronectin expression mediates the metastatic potential of NSCLC cells, with high expression levels of this protein associated to increased lung cancer metastasis^70^. Thus, we concluded that, overtime, A549 cells acquired metastatic-like properties when grown as ALI MCCs. Interestingly, the increased fibronectin expression was not linked to E-cadherin down-regulation (Fig. 3). In patients with NSCLC, impaired expression of functional E-cadherin is reported to be associated with tumour dedifferentiation^71,72^, lymph node metastasis^73^, and poor prognosis^74,75^. E-cadherin functional disruption is however not only caused by its down-regulation but also by many other mechanisms (e.g. decreased tyrosine phosphorylation of E-cadherin protein, down-regulation of α-/β-catenin)^71,76,77^. In accordance with the above, previous studies on clinical samples also evidenced that only 11.9% of the metastatic NSCLCs analysed over a cohort of 109 patients were E-cadherin-negative^78^. This might be reflected by our results showing that the mesenchymal features (*i.e*. fibronectin up-regulation overtime) were acquired by A549 cells forming ALI MCCs even without the loss of E-cadherin expression.

Regarding the ability of ALI multilayered mono-cultures to predict the efficacy of inhaled anticancer drugs, our first conclusion was that multilayered architecture conferred chemoresistance to the cell cultures (Fig. 4). This is consistent with data reported by other research groups, showing that drug efficacy is reduced in MCCs^38^ and in general when cancer cells are cultured at high density^79^. Since anticancer agents tend to be more active against actively proliferating cells, our data on the expression of the Ki-67 protein (Fig. 2), showing that A549 cells forming ALI MCCs moved into the resting (G_0_, Ki67-negative^80^) cell cycle phase overtime, suggested that the decrease in drugs’ efficacy could be due to the reduced proliferative activity of A549 cells in ALI MCCs grown for 14 d. Consistently with this hypothesis, previous studies have shown that (i) long-term culture of A549 cells induces a substantial modulation of cell cycle genes to result in a quiescent population^48^, and (ii) with increasing the number of layered cells, the percentage of Ki67-positive cells decreases^49^. Hence, the Ki67 protein downregulation detected in ALI MCCs was associated with the increasing thickness of the multilayered structure of the cultures as a function of time. Also, based on the knowledge available on diffusion gradients in MCCs^25,26^, we believe that in our experiments the multilayered structure of ALI MCCs acted as a physical barrier to drugs’ penetration, hindering them to reach their subcellular targets in the lower layers of the cultures and, subsequently, reducing the drugs’ efficacy. Similarly, cell cultures’ thickening could be responsible, in our study, for hindering nutrition diffusion gradients, with the downstream effect of triggering the cells population to shift into the G_0_ phase.

The chemoresistance of ALI multilayered mono-cultures was further proved in this study by monitoring the biochemical signatures of the cultures following drug exposure (Fig. 5). Most anti-cancer strategies currently used in clinical settings, including the chemotherapeutic agents tested herein, induce cancer cell death by activation of apoptosis signal transduction cascades (Supporting Figure S12)^81,82^. Failure to undergo apoptosis results in cancer cells’ drug resistance. Apoptosis is regulated through caspases activity. We tested the activity of ten caspases (from 1 to 10), evaluating the activation of both “apoptotic” (caspase-2, -3, -6, -7, -8, -9, and -10) and “pro-inflammatory” caspases (caspase-1, -4, and -5), which induce cell death by pyroptosis^83^. Our results confirmed that no apoptosis was triggered in ALI multilayered mono-cultures following drug treatment. Activation of caspases in response to anti-cancer chemotherapy can be initiated through activation of the extrinsic (receptor) pathway, or at the mitochondria, by stimulating the intrinsic pathway^81^. Lack of an increased caspases 1–10 activity excluded that the extrinsic apoptotic pathway was induced by drug exposure. It also suggested that the intrinsic pathway was not activated. To further validate this, the levels of cytochrome c in the cell cytoplasm were quantified, as a marker of mitochondrial outer membrane permeabilization (MOMP). No significant release of cytochrome c could be detected when ALI multilayered mono-cultures were exposed to vinblastine, cytarabine and methotrexate, as compared to untreated cultures (NT). This confirmed that the intrinsic apoptotic pathway was not activated by exposure to these three drugs. Conversely, significant release of cytochrome c could be detected under docetaxel treatment. The release of cytochrome c from mitochondria directly triggers procaspase-3 activation^84^. In our study, we found significantly low caspases 1–10 activity associated with docetaxel-exposure. Consistent with this result, no activation of procaspase-3 (into its cleaved form) could be detected in ALI multilayered mono-cultures exposed to docetaxel. Expression of the cleaved, active form of caspase-3 was lacking also in ALI MCCs exposed to vinblastine, cytarabine and methotrexate. It should be highlighted here that, reduced expression of cleaved caspase-3 is a hallmark of biological specimens derived from chemoresistant NSCLC patients^85^. Caspases cleave a number of different substrates in the cytoplasm, such as poly(ADP-ribose) polymerase (PARP), leading to cells’ apoptosis^82^. Our Western blot analysis showed that the lack in procaspase-3 cleavage was reflected by absence in PARP activation. Hence, this confirmed that ALI multilayered mono-cultures were also resistant to docetaxel, as well as the other three anti-cancer drugs. We therefore suggest that, in this *in vitro* model, A549 cells were able to overcome the mitochondrial damage and subsequent cytochrome c release induced by docetaxel treatment. In fact, it is known that cancer cells express high levels of the inhibitor of apoptosis proteins (IAPs), a feature that allows cells to block caspase activation initiated by a small amount of released cytochrome c^86^. As abovementioned, caspases 1–10 activity was reduced following docetaxel exposure. This further ruled out the release of Smac/DIABLO, which trigger caspases activation for exerting its pro-apoptotic functions^87^, following docetaxel-induced MOMP. Also, phosphorylation of p53 protein could not be detected upon exposure to any of the four drugs. Apoptotic signals triggered by chemotherapy can be transmitted via the phosphorylated form of the tumour suppressor p53 to mitochondria^88^, which in turn releases apoptogenic factors (cytochrome c and Smac/DIABLO). Thus, our results proved once again that ALI multilayered mono-cultures were resistant to the anti-cancer drugs tested. Finally, we found that the protein B-cell lymphoma-extra large (Bcl-xl) was expressed in untreated ALI multilayered cultures and was unaffected by drug exposure. Bcl-xl is an anti-apoptotic protein directly involved in inhibiting MOMP^89^. This strengthened our conclusion that ALI multilayered mono-cultures were chemoresistant to the four functionally and structurally different anti-cancer drugs tested. Such phenomenon of resistance to different classes of chemotherapeutics by cancer cells is generally referred to as “multidrug resistance” (MDR), and it leads to cancer relapse and death in patients. Our data proved that the MDR of ALI multilayered mono-cultures was driven by the MRP1/ABCC1 transporter (Fig. 8). This is consistent with the literature, showing that MRP1 levels are elevated in clinical samples of NSCLC^62,90^. A strong correlation has also been reported in clinics between high MRP1/ABCC1 expression levels and negative responses to various anti-cancer drugs^91–93^. Inhibition of MRP1/ABCC1 with reversan confirmed that the high MRP1/ABCC1 expression in ALI multilayered mono-cultures triggered chemoresistance to all four drugs tested.

Our second conclusion was that the chemoresistance of MCCs was independent of the culturing substrate used, and that MCCs grown in ALI conditions were less chemoresistant than their submerged equivalent (Fig. 4). To the best of our knowledge, this result is the first time to be reported, and no data can be found or is available in the scientific literature on the effects of ALI culturing conditions on drug sensitivity. Also, the ALI MCCs developed in this study demonstrated to be an equivalent alternative to 3D tumour spheroids for anti-cancer drug efficacy screening applications (Fig. 7). 3D tumour spheroids are the current gold standard in 3D alternative models for cancer research, and their potential for the efficacy screening of novel anti-cancer treatments has been largely demonstrated by the scientific community^94–96^. Chemo- and radio-cytotoxicity are the most important areas of use for 3D tumour spheroids^97,98^, as the clinical response to such treatments largely depends on parameters such as oxygen/nutrients gradients, compactness and pressure, apoptosis inhibition and permeability. These parameters can be modulated in 3D tumour spheroids to reproduce the *in vivo* scenario. Thus, in light of our expertise in the area^99^, we included in the study a valuable comparison of the chemoresistance of A549 cells forming ALI multilayered mono-cultures to that found in 3D tumour spheroids of the same cell line. We demonstrated that chemoresistance was generally comparable among the two *in vitro* models, with the main advantage of ALI MCCs over 3D tumour spheroids of being able to reproduce the direct contact of the lung epithelium with the gas phase.

Our third and final conclusion was that ALI MCCs may be a valid *in vitro* model to study and predict the efficacy of novel inhalable anti-cancer drugs in the future (Fig. 6). The nebulizer used within our study (Aeroneb® Pro nebuliser) is a clinically approved medical device, which has shown to have no impact on the quality (e.g. chemical structure and function) of the drug delivered^100^. It has also been validated for clinical and research use following the methacholine challenge testing (MCT) guidelines published by the European Respiratory Society^101^. Thus, we suggest that the decreased efficacy detected in ALI MCCs exposed by nebulization could be due to the poor water solubility of the drugs. The latter is one of the main serious limitations associated with the pulmonary delivery of chemotherapeutics. Most anti-cancer drugs cannot, in fact, be inhaled in their traditional form and require a special drug delivery system (e.g. carrier) to be deposited directly onto the lung epithelium. Supporting this need, our data showed that nebulized drugs were less effective in inducing cytotoxicity in ALI MCCs.

Therapeutic efficacy and testing of novel inhaled chemotherapeutics must ultimately be demonstrated in preclinical animal models prior to their clinical evaluation. However, the knowledge developed in this study, like the development and characterization of three-dimensional MCCs cultures of NSCLC, provides the *in vitro* tools capable of guiding the rational selection of inhaled anti-cancer candidates for animal testing, thus minimizing the number of animals used *per* study (principle of Reduction, “3Rs” framework). To achieve this, the tissue-mimetic model developed herein has demonstrated to increase the prediction efficiency of the current *in vitro* approaches by: (i) incorporating the necessary levels of biological complexity (3D architecture), (ii) achieving a “closer relevancy to the patient model” by reproducing MDR mechanisms observed in human NSCLC patients, and (iii) integrating culturing conditions at the Air-Liquid Interface that are compatible with aerosol administration methods. Also, the global valence of the presented preclinical model is its applicability to other sectors of the pharmaceutical and chemicals industries (e.g., toxicity assessment of inhaled compounds, products and nanomaterials), as a valid alternative to animal-based inhalation studies.

## Methods

### Cell culture

Human adenocarcinoma cells (A549 cell line) were obtained from the American Tissue Culture Collection (ATCC®) (LG Standards, England). The A549 cell line was authenticated using Short Tandem Repeat (STR) profiling (LGC Standards) showing that our A549 batch is an exact match for the ATCC® human cell line CCL-185 (A549) (100% match between the submitted sample and the database profile). A549 cell line-specific phenotypic responses (e.g. p21 expression in response to DNA damage) were confirmed as part of the laboratory GLP (Supporting Figure S13). A549 cells were cultured in Dulbecco’s Modified Eagle Medium (DMEM) (Gibco, Invitrogen, Bio-Sciences Ltd, Ireland) supplemented with glucose (1,000 mg/l), gentamicin (5 μg/ml) and 10% Fetal Bovine Serum (FBS) (Sigma-Aldrich, Ireland). Cells were cultured at 37 °C and 5% CO_2_. For cell seeding, cells were detached from cell culture flasks’ substrate with TryplE™ (Gibco, Invitrogen, Bio-Sciences Ltd, Ireland), centrifuged, counted using a Countess™ Automated Cell Counter (Invitrogen, Bio-Sciences Ltd, Ireland) and diluted in the supplemented culture medium at concentrations appropriate for each experiment. The seeding concentration of A549 cells was kept constant among all cell models (1.5 × 10^5^ cells/ml), with the exception of sub-confluent monolayered mono-cultures grown on plastic substrate and 3D spheroids, which were seeded at lower concentrations (5 × 10^3^ cells/ml and 1.25 × 10^5^ cells/ml, respectively).

### Sub-confluent monolayered mono-cultures

Sub-confluent monolayered mono-cultures were grown as per the experimental conditions indicated in the GDSC (Genomics of Drug Sensitivity in Cancer) database^56^. Briefly, A549 cells were seeded in 96-well plates (Nunc, Fisher Scientific, Ireland) in supplemented DMEM medium (final volume/well: 200 μl; seeding concentration: 3.1 × 10^3^ cells/cm^2^). Cells were cultured for 24 h at 37 °C and 5% CO_2_, obtaining sub-confluent monolayered cell cultures (15% cell confluence c.a., as determined by microscopic inspection).

### Submerged multilayered mono-cultures

A549 cells were seeded on 24-well plates (Costar, Corning Incorporated, Fisher Scientific, Ireland) (final volume/well: 500 μl) or on the apical side of Transwell™ Permeable Supports (Costar, Corning Incorporated, Fisher Scientific, Ireland) (final volume/support: 200 μl). The cell concentration per cm^2^ was equal to 1.5 × 10^5^ cells/cm^2^ in both *in vitro* models. The Transwell™ supports were formed by polyethylene terephthalate (PET) membrane inserts of 6.5 mm of diameter (growth area: 0.33 cm^2^) and pore size of 0.4 μm, attached onto hanging supports fitting 24-well plates. In multi-layered mono-cultures grown on PET, 700 μl supplemented DMEM medium was also added to the basolateral chamber. Cultures were grown for 14 d and both apical and basolateral media changed every 3 d.

### ALI multilayered mono-cultures

700 μl supplemented DMEM medium was added to the wells of 24-well plates, and Transwell™ Permeable Supports were inserted into the wells. A549 cells were added to the apical compartment of the Transwell™ supports (final volume/support: 200 μl; cell concentration: 1.5 × 10^5^ cells/cm^2^) and incubated for 24 h at 37 °C and 5% CO_2_ to allow cell attachment to the membrane. After 24 h, the media in the apical compartment was removed, leaving A549 cells in direct contact with the gas phase, at the Air-Liquid Interface (ALI). The ALI multilayered mono-cultures were cultured for up to 14 d and medium in the basolateral chamber was changed every 3 d.

### 3D tumour spheroids

Spheroids of A549 cells were formed by means of the GravityPLUS^TM^ Hanging Drop System (InSphero Europe GmbH, Perkin Elmer, Ireland), following the manufacturer’s protocol. Briefly, A549 cells were suspended in supplemented DMEM medium and 40 μl of cell suspension was added to each well of the GravityPLUS^TM^ plate, forming a drop (5,000 cells/drop c.a.). After 3 d at 37 °C and 5% CO_2_, cell medium was changed by aspirating 20 μl from each well and delivering 20 μl of fresh supplemented DMEM medium with a pipette. 3D spheroid formation was assessed by inverted microscopy. At t = 4 d, 3D spheroids were transferred to the raster plate provided in the GravityPLUS^TM^ Hanging Drop System by slowly adding 70 μl of fresh medium to each well. Successful transfer was validated through microscopic inspection of the wells using an inverted microscope. 3D spheroids were then cultured up to 14 d. Cell medium was changed every 2 d.

### Characterization of the *in vitro* models

#### Cell viability and cytotoxicity responses

A panel of commercially available assays was used to screen the cell viability and cytotoxic responses of the cultures. The assays used have been previously reported to be suitable for screening complex 3D cultures^99,102^.

#### Quantification of the percentage of live A549 cells

At each time-/end-point of interest, the percentage of live cells was quantitatively determined by means of BD Accuri® C6 flow cytometer (Becton Dickinson Biosciences, Oxford, UK). Cell cultures were disaggregated by TryplE™ (10 min, 37 °C) and/or by pipetting vigorously, and A549 cells were stained with the LIVE/DEAD Fixable Red Dead Cell Stain Kit (Invitrogen, Bio-Sciences Ltd, Ireland) for 30 min, protected from light. When studying ALI multi-layered co-cultures, only cells growing on the apical side of the Transwell™ supports (A549 cells) were analysed. Cells were then centrifuged at 3,000 rpm for 1 min and fixed with 3.6% formaldehyde (FA) (Sigma-Aldrich, Ireland) for 15 min (ambient temperature). Following centrifugation (3,000 rpm; 1 min), the cell pellet was re-suspended in 1% bovine serum albumin (BSA) (Sigma-Aldrich, Ireland) in PBS and analysed using the blue laser (λ_excitation_ = 488 nm) and the 585/40 nm filter. Cell population analysis was performed as per the manufacturer’s protocol. Briefly, the cells were visualized using the forward scatter (FSC-A) versus red fluorescence intensity (FL2-H) scatter plot and a gate was applied to determine the percentage of live and dead cells (Supporting Figure S14). 100 µl of cell suspension was analysed for each sample. Measurements for each sample were carried out in duplicate to ensure data reliability. Two replicates of the same sample were included in each test (n_replicates_ = 2), and experiments repeated three times (n_tests_ = 3). Results are presented as average ± standard error of the mean.

Quantitative results were confirmed by Laser Scanning Confocal Microscopy (LSCM) inspection of the live specimens stained with Hoechst 33342 and ethidium homodimer-1 (Eth-1) (Invitrogen, Bio-Sciences Ltd, Ireland) (40 min, ambient temperature).

#### Quantification of ATP levels

At the time-/end-points of interest, the CellTiter Glo® 3D Reagent (Promega, MyBio, Ireland) was added to the cultures and incubated for 30 min on a plate shaker (ambient temperature). Supernatants were then transferred to 96-well clear bottom black microplates (Corning, Fischer Scientific, Ireland) and luminescence read by an FLx800 plate reader (BioTek, Mason Technology, Ireland) (integration time = 0.5 s/well; gain = 135). Each time-/end-point was tested in duplicate (n_replicates_ = 2), and experiments repeated three times (n_tests_ = 3). Results are presented as average ± standard error of the mean.

#### Lactate dehydrogenase (LDH) cytotoxicity assay

Supernatants were harvested at the time- and end-point under investigation, and cytotoxicity was evaluated by Thermo Scientific Pierce LDH Cytotoxicity Assay Kit (Fisher Scientific, Ireland), as per manufacturers’ protocols. Untreated cultures and *in vitro* models exposed to LDH Lysis Buffer (1× in supplemented medium) for 45 min at 37 °C were included in the experimental design as negative (NT) and positive (PT) controls, respectively. An Epoch microplate reader (Biotek, Mason Technologies, Ireland) was used to read the absorbance values at wavelengths (λ) equal to 490 and 680 nm. The LDH activity was calculated as for Equation (1); whereas, the percentage (%) cytotoxicity was extrapolated from Equation (2).

1 $$LDH\,activity=Absorbanc{e}_{\lambda =490nm}-Absorbanc{e}_{\lambda =680nm}$$

2 $$Cytotoxicity\,( \% )=\frac{LDH\,activit{y}_{Treatedculture}-LDH\,activit{y}_{NT}}{LDH\,activit{y}_{PT}-LDH\,activit{y}_{NT}}$$

Each time-/end-point was tested in duplicate (n_replicates_ = 2), and experiments were repeated three times (n_tests_ = 3). Data are presented as average ± standard error of the mean.

#### Lucifer Yellow (LY) permeability assay

The crossing of LY (Sigma-Aldrich, Ireland) from the apical to the basolateral compartment of ALI multilayered mono-cultures was used to investigate the confluency and integrity of the epithelial layer, as described by Dekali *et al*.^103^. Medium in the basolateral chamber was removed, and 700 μl of pre-warmed Hanks’ Balanced Salt Solution (HBSS) (Gibco, Thermo Fisher, Fisher Scientific, Ireland) was added. 200 μl of LY solution (400 μg/ml) in HBSS was added to the apical side of the cultures. After 1 h incubation in humidified atmosphere at 37 °C and 5% CO_2_, fluorescence was measured in the basolateral compartment using an FLx800 plate reader (λ_excitation_ = 485/20 nm; λ_emission_ = 528/20 nm). The concentration of LY was extrapolated based on a standard curve. The percentage (%) of LY passage was then calculated based on Equation (3), where C_B_ is LY concentration in the basolateral compartment as determined experimentally, V_B_ the volume in the basolateral compartment (700 μl), C_0_ the initial LY concentration in the apical compartment (400 μg/ml) and V_A_ the volume in the apical compartment (200 μl).

3 $$ \% \,LY\,passage=\frac{{C}_{B}\times {V}_{B}}{{C}_{0}\times {V}_{A}}\times 100$$

Finally, the apparent permeability coefficient (P_app_) was determined based on Equation (4). In the latest, t refers to time (equal to 1 h in our experimental design), while A corresponds to the surface area of the filter (0.3 cm^2^).

4 $${P}_{app}=(\frac{{C}_{B}}{t}\times {V}_{B})\times \frac{1}{A\times {C}_{0}}$$

### Exposure to anti-cancer drugs

Cell cultures were exposed to four chemotherapeutic drugs: anhydrous docetaxel, vinblastine sulphate, cytarabine and methotrexate (Sigma-Aldrich, Ireland). Selection criterion was the efficacy in inducing A549 cells death based on the GDSC database. Docetaxel was the most active drug tested, while methotrexate was the less effective in inducing cancer cells death. Drugs were purchased as in the form specified by the European Pharmacopoeia. *In vitro* models were exposed to drugs for 72 h in duplicate (n_replicates_ = 2). Experiments were repeated three times (n_tests_ = 3).

#### Control experiment (GDSC database experimental conditions)

To validate the sensitivity to chemotherapy of our A549 cells batch, sub-confluent monolayer mono-cultures were exposed to the four anti-cancer drugs at a concentration equal to the nominal half-maximal inhibitory concentration (IC_50_) reported for these anti-cancer agents by the GDSC database for A549 cells. These are: 1 × 10^−3^, 1 × 10^−2^, 0.13 and 11.39 μM for docetaxel, vinblastine, cytarabine and methotrexate, respectively. Drugs were diluted in supplemented DMEM medium and added to the cultures with a pipette (direct inoculation) following supernatants removal. Cells viability was measured after 72 h exposure using a fluorescence-based assay. Briefly, cells were first fixed with 3.6% FA for 30 min and then stained with the fluorescent DNA stain Hoechst 33342 (1 µg/ml) for 1 h. The fluorescent signal intensity was quantified by an area scan for each well by means of FLx800 plate reader (λ_excitation_ = 360/40 nm; λ_emission_ = 460/40 nm). Cell cultures exposed to supplemented DMEM medium were used as negative controls (NT). Fluorescence intensity data were normalized on the negative control (NT) read-outs.

#### Exposure by direct inoculation (pipetting)

3D tumour spheroids and submerged multilayered mono-cultures were exposed to the four drugs diluted in supplemented DMEM medium. Supernatants were first removed from these cultures, and drug-containing medium (70 μl in 3D spheroids and 200 μl in submerged multilayered cultures) was then added to the *in vitro* models *via* pipetting.

For exposing ALI multilayered mono-cultures, drugs were dispersed in physiological hypertonic saline, constituted by a 0.9% NaCl solution supplemented with 1.25 mM CaCl_2_ and 10 mM HEPES (N-2-hydroxyethylpiperazine-N-2-ethane sulfonic acid) (all purchased from Sigma-Aldrich, Ireland). Such solution is a fully biocompatible vehicle, ensuring that no cytotoxic response was triggered by the vehicle itself. Drugs were administered by pipette to the apical side of the ALI cultures. In inhalation therapy, drugs are deposited as aerosols onto the air-facing lung epithelium. To test a physiological drug application scenario, a small volume (30 μl) of drugs solution was added to the apical side of the cultures, as previously reported^47^, to ensure the direct contact of the epithelium with the gas phase.

Cell cultures exposed to saline or supplemented DMEM medium were included as negative controls (NT) in the experimental design, as appropriate.

#### Nebulization (Aeroneb® Pro nebuliser)

A small-volume nebulizer based on vibrating-mesh technology (Aeroneb® Pro nebuliser, Aerogen Ltd, Galway, Ireland) was used for exposing ALI multilayered mono-cultures. The Aeroneb® Pro nebuliser, which is currently in use in clinical settings, can be easily adapted for the delivery of liquid aerosol on cells cultured in ALI conditions. Aerosol-to-cell delivery proved in fact to be reproducible and comparable to direct inoculation in experiments using aerosolized fluorescein as surrogate drug (Supporting Figure S7). A small volume (30 μl) of drug liquid aerosol was delivered to each ALI multilayered mono-culture to mimic more closely the *in vivo* administration conditions^104^. The high ionic strength of the physiological saline used as drug vehicle ensured optimal flow output. One drug dose was tested for each drug. This was equal to their nominal IC_50_ concentration.

#### Cell response to drug exposure

The percentage of live A549 cells, as well the cytotoxicity, following drug exposure were quantified by flow cytometry and LDH cytotoxicity assay, as described in sections 4.2.1.

#### Caspases 1–10 activity assay

The CasPASE^TM^ Apoptosis Colorimetric Assay (G-Biosciences, VWR International, Ireland) was used to evaluate the activity of caspases 1–10, which are key early indicators of apoptosis, in A549 cells following exposure to docetaxel, vinblastine, cytarabine and methotrexate. Drugs were tested at their nominal IC_50_ concentration, as for GDSC database. ALI MCCs were exposed by direct inoculation. Cell lysates were obtained by adding 200 μl of chilled CasPASE^TM^ Lysis Buffer (G-Biosciences, VWR International, Ireland) to ALI multilayered mono- and co-cultures, followed by 5 cycles of freezing and thawing. Untreated cultures were also lysed as negative control (NT). Assay was carried out as per manufacturer’s protocol, and absorbance was determined at λ = 405 nm by means of an Epoch microplate reader. The units of caspase activity were calculated using Equation 5:

5 $$units\,caspase\,activity=\frac{{\rm{\Delta }}O{D}_{sample}/{\rm{\Delta }}O{D}_{blank}}{minute}\times {(calibrationcurveslope)}^{-1}$$

where ΔOD is the rate of increase in optical density (OD) for each sample or for the blank (*i.e*., the CasPASE^TM^ Assay Buffer).

#### Cytochrome C release from mitochondria

Levels of cytochrome C in the cell cytoplasm of A549 cells forming ALI multilayered mono-cultures were quantified by Enzyme ImmunoSorbent Assay (ELISA) (Cytochrome c ELISA Kit, Invitrogen, Biosciences Ltd, Ireland), following the manufacture’s protocol. ALI MCCs were exposed for 72 h to docetaxel, vinblastine, cytarabine and methotrexate. Drugs were tested at their nominal IC_50_ concentration, as for GDSC database, and were added to the cultures by direct inoculation. Untreated cultures were also tested as negative control (NT). Detection of cytochrome c released from the mitochondria to the cytosol was achieved by selective lysis of the cell membrane, using a Cell Extraction Buffer (Invitrogen, Biosciences Ltd, Ireland), supplemented with protease inhibitor cocktail and phenylmethylsulfonyl fluoride (PMSF) (both from Santa Cruz Biotechnology Inc., Fannin Limited, Dublin, Ireland). For assay read-outs, the optical density of each well at λ = 450 nm was determined using an Epoch microplate reader.

#### Inhibition of chemoresistance

ALI multilayered mono-cultures were exposed by direct inoculation to nine concentrations of docetaxel (Table 1) (ten-fold dilution series over a 10^8^-fold concentration range) in the absence and presence of the inhibitor reversan (10 μM) (Santa Cruz Biotechnology, Ireland). Reversan was dispersed in drug-containing hypertonic saline at the desired concentration.

**Table 1:** Concentrations of docetaxel tested in this study to evaluate the specific mechanism of chemoresistance in ALI multi-layered mono-cultures.

Concentrations tested (μM)
Docetaxel
1 × 10^−7^
1 × 10^−6^
1 × 10^−5^
1 × 10^−4^
1 × 10^−3^ (nominal IC_50_)
1 × 10^−2^
0.1
1
10

### Techniques

#### Laser Scanning Confocal Microscopy (LSCM)

LSCM was used to assess F-actin organization, Ki67 protein expression and hypoxia detection. LSCM imaging was carried out by means of a ZEISS 510 Meta confocal microscope equipped with a Zeiss Zen software (Carl Zeiss, Axiovert, Germany). Series of z-stack images were acquired and then analysed by ImageJ software.

For staining F-actin and Ki67 protein, A549 cells were fixed with 3.6% FA for 10 min at ambient temperature, permeabilized with 0.25% Triton X-100 in PBS for 10 min and incubated with 5% BSA in PBS for 1 h (blocking step). Specimens were then stained with Hoechst 33342 (1 µg/ml) for nuclei, rhodamine phalloidin (1:50) for F-actin or Mouse anti-human Ki-67 (FITC) (1 µg/ml) for Ki67 expression (all supplied by Invitrogen, Fisher Scientific, Ireland). The staining solutions were prepared in 1% BSA in PBS. For cultures grown on Transwell^TM^ supports, solutions were added in both apical and basolateral compartment. The specimens were incubated at ambient temperature for 3 h in the dark and rinsed with PBS.

When monitoring hypoxia in 3D tumour spheroids, live specimens were stained with Image-iT® Hypoxia Reagent (1:100 in fresh medium) (Invitrogen, Bio-Sciences Ltd, Ireland) and Hoechst 33342 (1 µg/ml) for 30 min in the dark, at ambient temperature. Specimens were then immediately imaged by LSCM.

For imaging purposes, fixed specimens were mounted on glass slides in transparent mounting medium (VECTASHIELD, Vector Laboratories Inc., CA, USA). PET membranes were detached from the plastic support with a scalpel blade. Fixed 3D tumour spheroids were carefully transferred to glass slides by means of a pipette; whereas, live specimens were imaged in their growing environment.

#### Trans-Epithelial Electrical Resistance (TEER)

TEER measurements were performed on ALI multilayered mono-cultures by means of an epithelial voltmeter (EVOM^2^, World Precision Instruments Inc., Hertfordshire, UK) that produces an AC current, calibrated according to manufacturer’s instruction. 200 μl of pre-warmed PBS was added on the apical side of the cultures to allow measurements, which were carried out in triplicate for each sample. TEER of cell-free inserts was also measured as a baseline. Experiments were repeated three times (n_tests_ = 3) with 2 replicates (n_replicates_ = 2). TEER values are expressed as Ohms (Ω)×cm^2^ and are calculated according to Equation (6) (insert area = 0.3 cm^2^). Results are presented as average ± standard error of the mean.

6 $$TEER=({{\rm{\Omega }}}_{ALIculture}-{{\rm{\Omega }}}_{cell-freeinsert})\times insert\,area$$

#### Cell lysis, SDS-PAGE and Western immuno-blotting

Cell cultures were washed with ice-cold PBS. RIPA buffer (Santa Cruz Biotechnology Inc., Fannin Limited, Dublin, Ireland) supplemented with sodium orthovanadate (Santa Cruz Biotechnology Inc., Fannin Limited, Dublin, Ireland), protease inhibitor cocktail and PMSF was used as lysis buffer. Supplemented RIPA buffer was added to the apical compartment of the Transwell™ supports, and A549 cells scraped to favour cell lysis. 3D spheroids were transferred to Eppendorf tubes containing supplemented RIPA buffer and placed on ice. In this instance, vigorous pipetting was used to ensure complete cell lysis. Following sonication for 10 min in a sonic bath to favour cell lysis, all lysates were centrifuged for 15 min at 15,000 rpm at 4 °C. The protein content of each lysate obtained was quantified using the Pierce BCA Protein Assay Kit (Product no 23225; Thermo Scientific, Fisher Scientific, Ireland), as per manufacturer’s protocol.

For SDS-PAGE, appropriate volumes of lysates were diluted in supplemented RIPA lysis buffer and NuPAGE® LDS Sample Buffer supplemented with NuPAGE® Sample Reducing Agent (both supplied by Thermo Scientific, Fisher Scientific, Ireland). Samples were heated for 10 min at 70 °C, and resolved on pre-casted NuPAGE® 4–12% Bis–Tris Gels (Novex®, Life Technologies, Fisher Scientific, Dublin, Ireland) at 200 V. NuPAGE® MES (2-(N-morpholino) ethane sulfonic acid) SDS or MOPS (3-(N-morpholino) propane sulfonic acid) SDS Running Buffer (Thermo Fisher Scientific, Fischer Scientific, Ireland) were used to resolve proteins with molecular weights ranging from 200 to 2 kDa and 260 to 14 kDa, respectively. A biotinylated protein ladder (Cell Signaling Technology Inc, Brennan & Company, Ireland) was also resolved within the same gel as a control. Resolved proteins and molecular weight marker were electrophoretically transferred to polyvinylidene difluoride membranes (Immobilion P transfer membrane, Merck Millipore, Ireland) by wet transfer for 2 h at 30 V. Polyvinylidene difluoride membranes were then blocked in either 5% non-fat dry milk in TBS-T 1 × (0.1% Tween20 in TBS) or 5% BSA TBS-T 1 × for 1 h at RT. TBS 10 × was purchased from Santa Cruz Biotechnology (Ireland), diluted in DI water or 0.1% Tween20-DI water to obtain TBS 1× and TBS-T 1×, respectively. Staining with primary antibodies (Table 2) was carried out overnight at 4 °C. GADPH, β-actin or α-tubulin protein bands were used as loading controls. The membranes were then washed and incubated with their respective HRP-linked secondary antibodies (anti-rabbit IgG HRP-linked antibody or anti-mouse IgG HRP-linked antibody, both from Cell Signalling Technology Inc, Brennan & Company) (1 h, RT, with gentle agitation). Anti-biotin, HRP-linked Antibody (Cell Signalling Technology Inc, Brennan & Company, Ireland) was used to stain the protein ladder. Probed membranes were then washed, incubated with HRP substrate (Luminata^TM^ Forte Western HRP Substrate, Merck Millipore, Ireland) and protein bands visualised by chemiluminescent detection on CL-XPosure Film (Thermo Scientific, Fisher Scientific, Ireland). Relative proteins’ expression levels were quantified by ImageJ software.

**Table 2:** Primary antibodies used for Western blotting analysis in this study.

Antibody	Dilution	Diluent
p21 Waf1/Cip1 (12D1) Rabbit mAb	1:1000	5% BSA in TBS-T 1×
Phospho-p53 (Ser15) (16G8) Mouse mAb	1:500	5% BSA in TBS-T 1×
Mouse anti-human fibronectin N-terminal monoclonal antibody	1:1000	5% BSA in TBS-T 1×
MDR1/ABCB1 (D3H1Q) Rabbit mAb	1:1000	5% BSA in TBS-T 1×
Vimentin (D21H3) XP® Rabbit mAb	1:1000	5% BSA in TBS-T 1×
Phospho-SMAD2 (Ser465/467) (138D4) Rabbit mAb	1:1000	5% BSA in TBS-T 1×
β-Actin Antibody	1:1000	5% BSA in TBS-T 1×
Caspase-3 Antibody	1:1000	5% non-fat dry milk in TBS-T 1×
Cleaved Caspase-3 (Asp175) (5A1E) Rabbit mAb	1:1000	5% non-fat dry milk in TBS-T 1×
PARP (46D11) Rabbit mAb	1:1000	5% non-fat dry milk in TBS-T 1×
MRP1/ABCC1 (D7O8N) Rabbit mAb	1:1000	5% non-fat dry milk in TBS-T 1×
Bcl-xl (54H6) Rabbit mAb	1:1000	5% non-fat dry milk in TBS-T 1×
E-Cadherin (4A2) Mouse mAb	1:1000	5% non-fat dry milk in TBS-T 1×
GADPH (D16H11) XP® Rabbit mAb	1:1000	5% non-fat dry milk in TBS-T 1×

Antibodies dilutions and the diluent in which they were prepared are also specified. With the exception of Anti-Surfactant protein D antibody [12G5] (Abcam, Ireland) and mouse anti-human fibronectin N-terminal monoclonal antibody (Millipore Merck, Ireland), all antibodies were purchased from Cell Signaling Technology Inc. (Brennan & Company, Ireland).

### Statistical analysis

Graph-Pad Prism (Graph-Pad Software Inc., La Jolla, CA, USA) was used to carry out the statistical analysis. A *p* value < 0.05 was considered statistically significant. The statistical tests used for each dataset are specified in the corresponding figure caption.

## Electronic supplementary material

Supplementary Information

Video S1

## Data Availability

All data generated or analysed during this study are included in this article. The raw datasets generated during and/or analysed during the current study are available from the corresponding author on reasonable request.
