# Supplementary material for: Multilayered Cultures of NSCLC cells grown at the Air-Liquid Interface allow the efficacy testing of inhaled anti-cancer drugs
Source: Sci Rep. 2018 Aug 27;8:12920. doi: 10.1038/s41598-018-31332-6 (PMC6110800; doi:10.1038/s41598-018-31332-6)
Supplement: Supplementary file 1 — Supplementary Information [file 41598_2018_31332_MOESM1_ESM.pdf]

Multilayered Cultures of NSCLC cells grown at the Air-Liquid Interface allow the efficacy testing of inhaled anti-cancer drugs

Dania Movia[1,*], Despina Bazou[2], Yuri Volkov[1,3,4], Adriele Prina-Mello[1,3]

[1]Department of Clinical Medicine/Trinity Translational Medicine Institute (TTMI), Trinity College Dublin, Ireland

[2]Mater Misericordiae University Hospital, Dublin, Ireland

[3]AMBER Centre, CRANN Institute, Trinity College Dublin, Ireland

[4]Department of Histology, Cytology and Embryology, First Moscow State Sechenov Medical University, Russian Federation

- Supporting Information -

SUPPORTING FIGURES

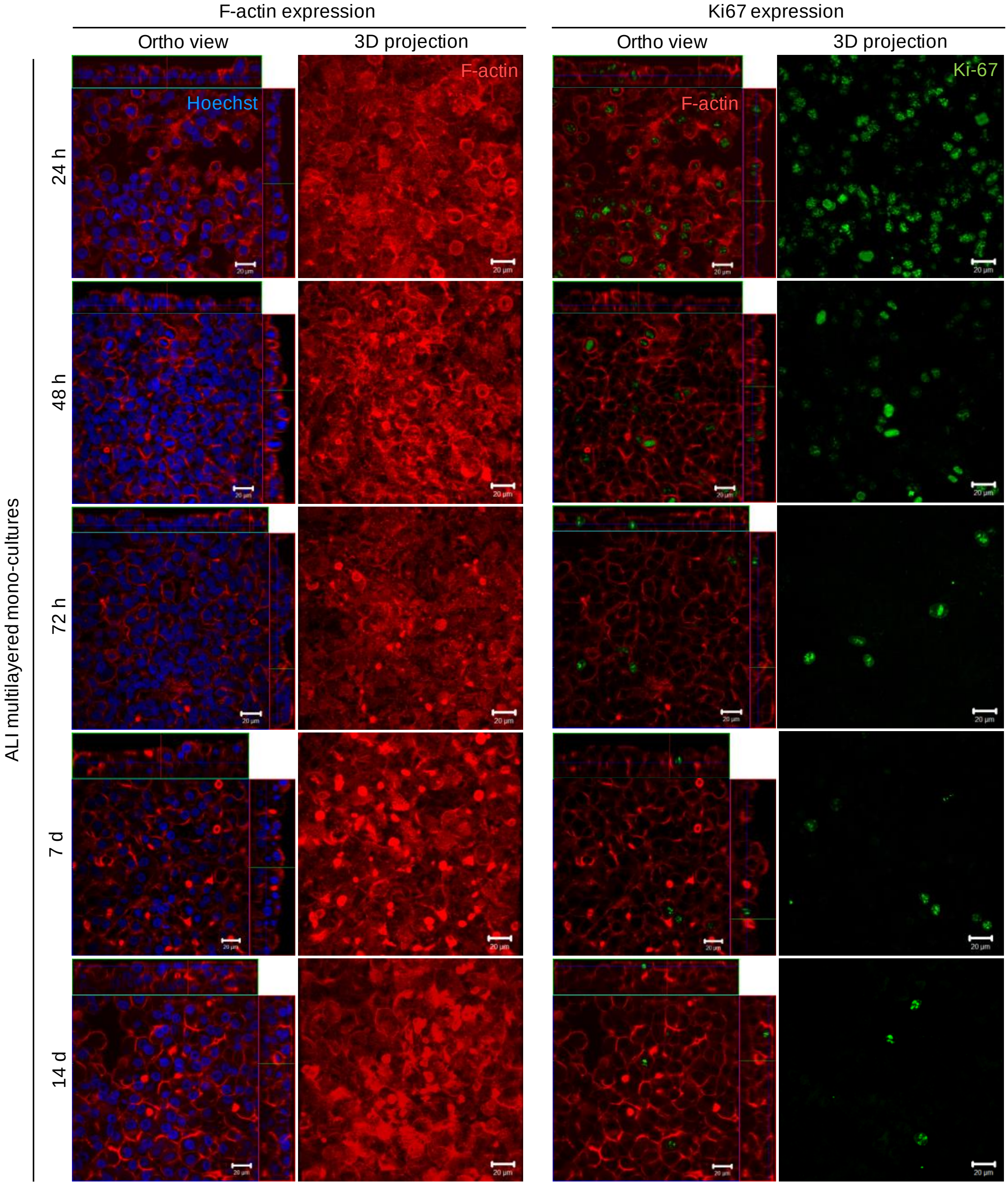

Figure S1. Representative LSCM images of the (from left to right) F-actin organization (in red) and Ki67 protein expression (in green) in ALI multilayered mono-cultures grown for 24 h, 48 h, 72 h, 7 d and 14 d. Cell nuclei were also stained with Hoechst 33342 (in blue). Z-stack images were acquired and are here presented in orthogonal view (ortho view) or as three-dimensional projections. Scale bars: 20 μm (objective lens: 63×).

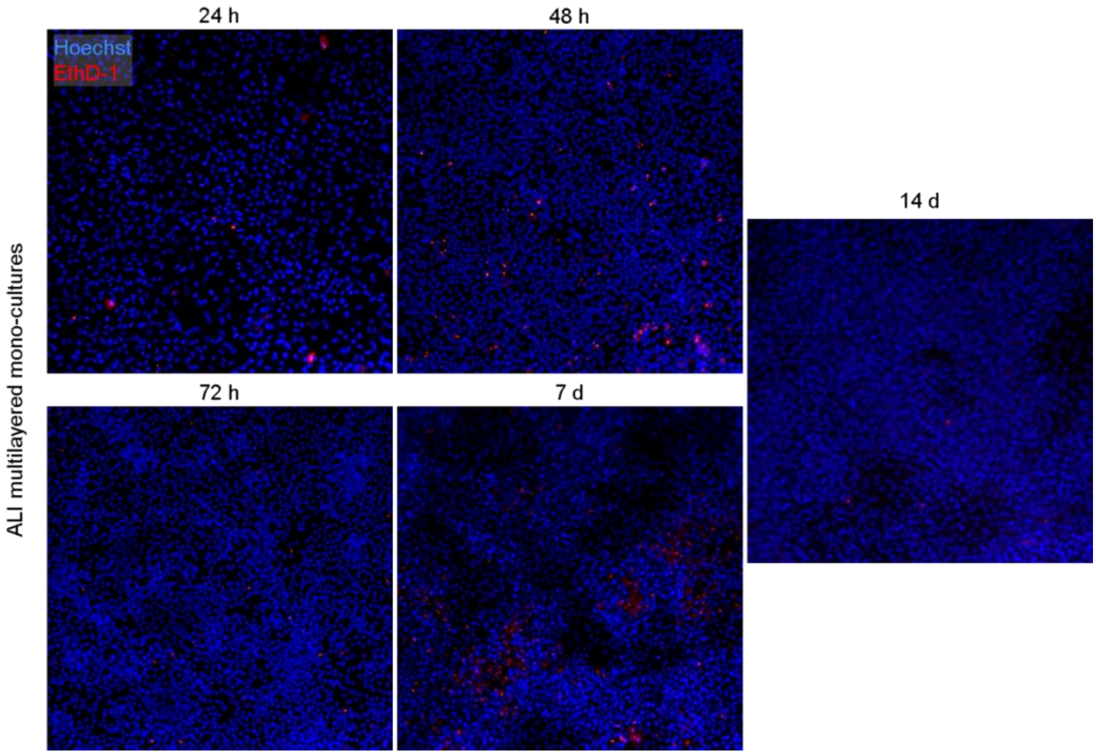

Figure S2. Representative LSCM images of ALI multilayered mono-cultures grown for 24 h, 48 h, 72 h, 7 d and 14 d and stained with Hoechst 33342 for cell nuclei (in blue) and EthD-1 for dead cells (in red). Z-stack images were acquired and are here presented in projection mode. Objective lens: 20×.

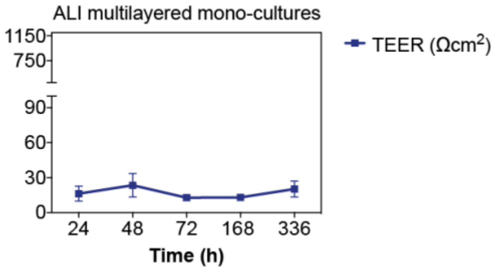

Figure S3. Time-dependent changes in TEER in ALI multilayered mono-cultures grown up to 14 d. Data are shown as average ± standard error of the mean ($n_{replicates}$ = 2; n_{tests} = 3). Changes were not statistically significant (two-way ANOVA and Bonferroni post-test).

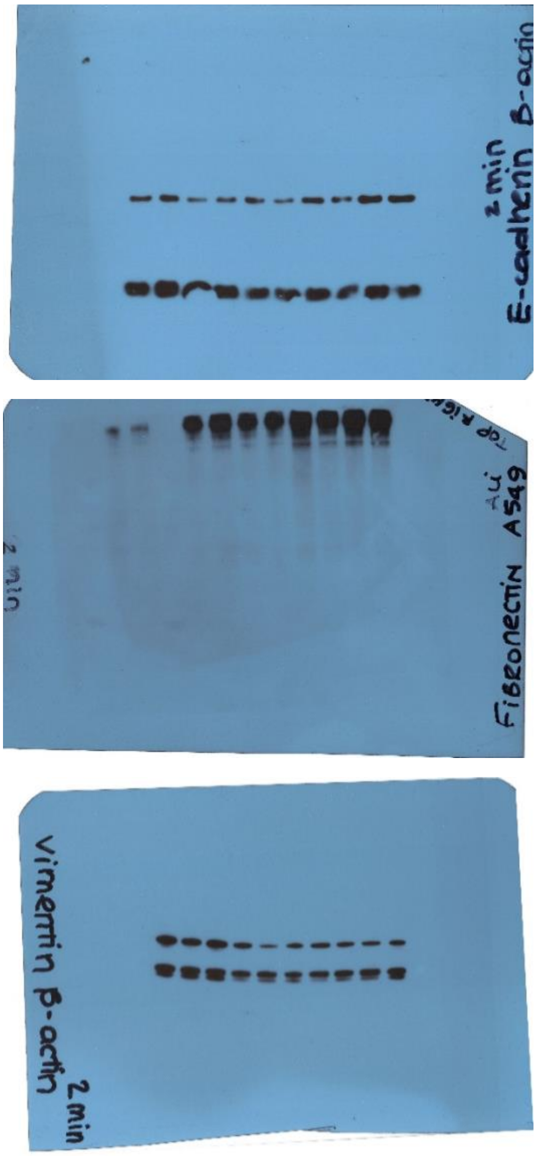

Figure S4. Original full-length Western blots showing the expression of (from top to bottom) E-cadherin (epithelial marker), fibronectin and vimentin (mesenchymal markers) in A549 cells forming ALI multilayered mono-cultures and cultured up to 14 d.

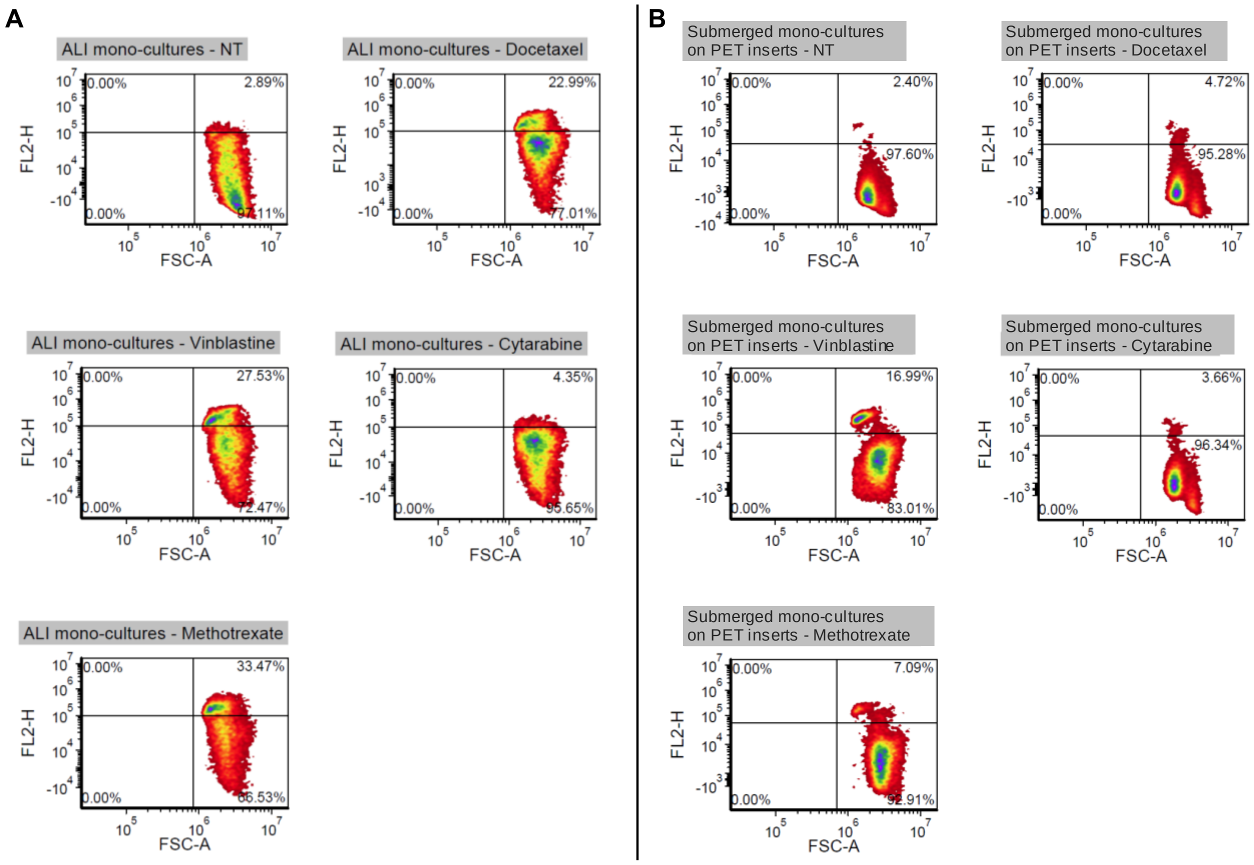

Figure S5. Scatter plots showing the percentage (%) of live and dead A549 cells detected in multilayered mono-cultures grown on the PET inserts of Transwell™ supports in (A) ALI or (B) submerged conditions, by means of flow cytometry. MCCs were exposed by direct inoculation to four different chemotherapeutics (docetaxel, vinblastine, cytarabine and methotrexate) at their nominal IC_{50} concentration for 72 h. Untreated MCCs were also analysed as negative control (NT). Counted live and dead cells can be visualised in the bottom right and top right quadrants, respectively. Data shown are representative of the results collected for multiple replicates run in the study.

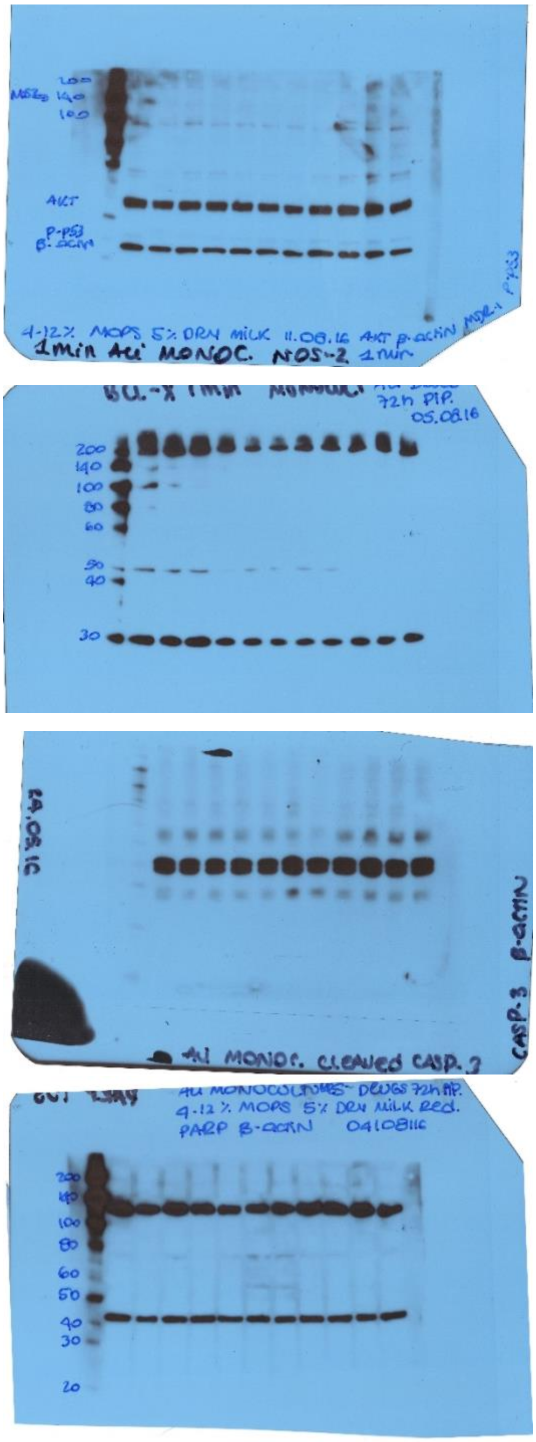

Figure S6. Original full-length Western blots showing the expression of (from top to bottom) phospho-p53 (p-p53), Bcl-xl, procaspase-3 and caspase-3, PARP and its cleaved form (cleaved PARP) in A549 cells cultured as ALI multilayered mono-cultures for 14 d and then exposed to docetaxel (Doc), vinblastine (Vin), cytarabine (Cyt) or methotrexate (Met) at their nominal IC50 for 72 h. Cell cultures were exposed to drugs by direct inoculation.

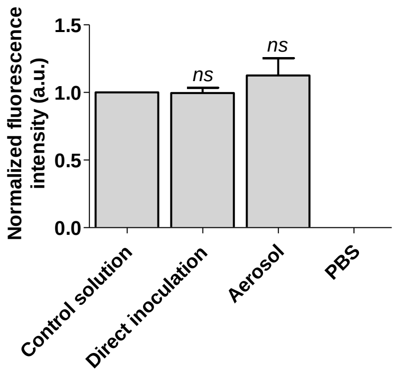

Figure S7. The drug delivery efficiency of the Aeroneb® Pro nebuliser was characterized by assaying the aerosolized delivery of fluorescein, as a surrogate drug, into three Transwell inserts. A 1 mg/ml solution of fluorescein sodium salt (Sigma-Aldrich, Ireland) dissolved in PBS (referred to as stock solution) was aerosolized for 15 seconds to a Transwell insert (0.4 µm pore size) of a standard 24-well plate (total volume delivered: 30 µl). 170 µl of PBS was added to the insert, the resulting solution transferred to a 96-well plate, and its fluorescence quantified by a FLx800 plate reader ($\lambda_{excitation}$ = 485/20 nm; $\lambda_{emission}$ = 528/20 nm; Biotek, Mason Technologies, Ireland). Fluorescence read-outs were compared to that of: 1) PBS (as baseline); 2) 30 µl of the stock solution diluted in 170 µl of PBS (control solution); and 3) 30 µl of the stock solution added to three inserts with a pipette, where 170 µl of PBS was added, and transferred to a 96-well plate (direct inoculation). No significant differences could be detected between the fluorescence intensities of the various samples (t-test). As expected, PBS did not show any fluorescence. Data are shown as average ± standard deviation ($n_{replicates}$ = 3) and are normalized on the fluorescence of the control solution.

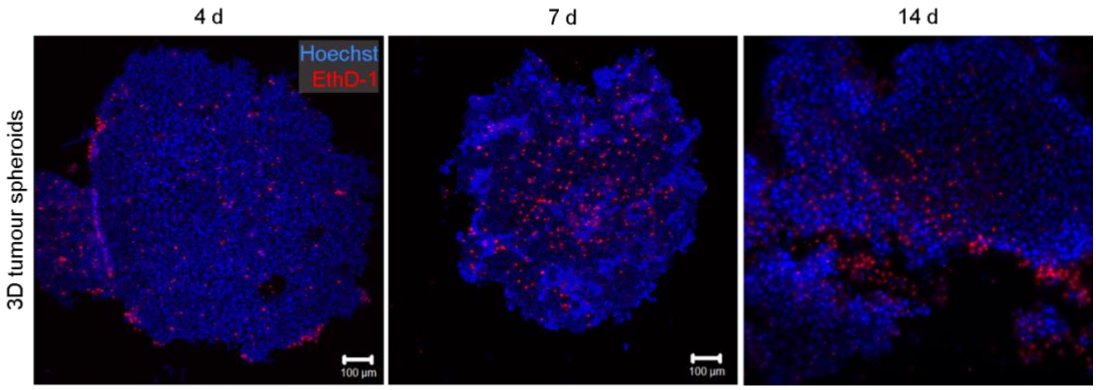

Figure S8. Representative LSCM images of 3D tumour spheroids grown for 4, 7 and 14 d. Live spheroids were stained with Hoechst 33342 for cell nuclei (in blue) and EthD-1 for dead cells (in red). Z-stack images were acquired and are here presented in projection mode. Objective lens: 20×.

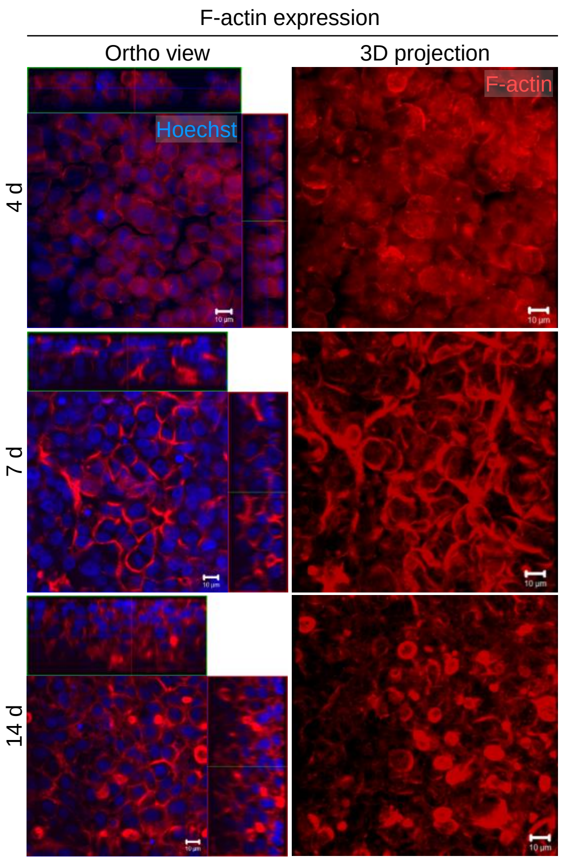

Figure S9. Representative LSCM images of the F-actin organization (in red) in 3D tumour spheroids of A549 cells grown for 4, 7 and 14 d. Cell nuclei were also stained with Hoechst 33342 (in blue). Z-stack images were acquired and are here presented in orthogonal view (ortho view) or as three-dimensional (3D) projections. Scale bars: 20 µm (objective lens: 63×).

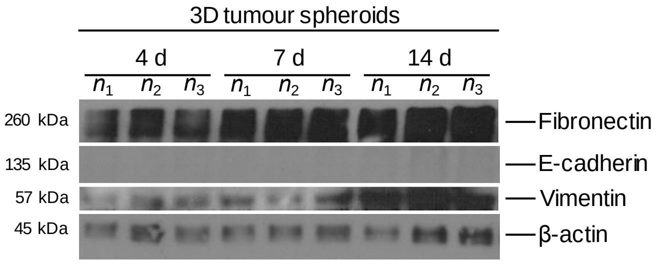

Figure S10. Western blot analysis of E-cadherin (epithelial marker), vimentin and fibronectin (mesenchymal markers) in A549 cells cultured as 3D tumour spheroids up to 14 d. The time-points examined were: 4, 7 and 14 d. Abbreviations "n_1", "n_2" and "n_3" indicate different replicates. β-actin expression is also reported as proteins loading control.

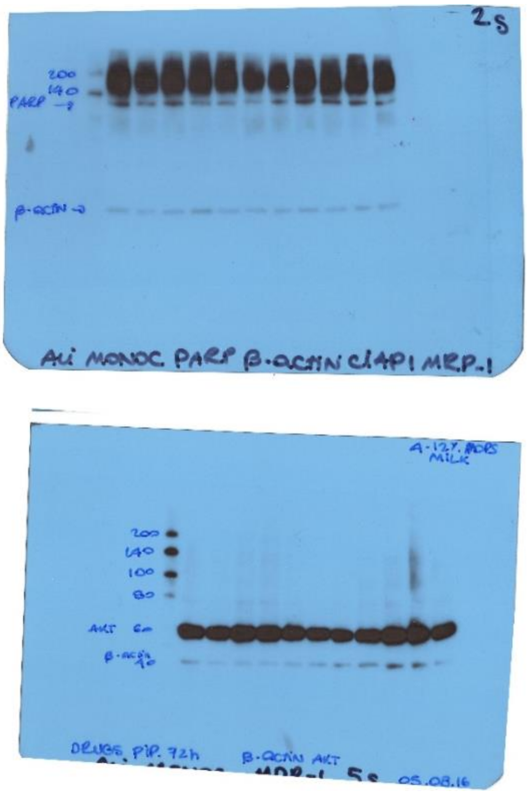

Figure S11. Original full-length Western blots showing the expression of (from top to bottom) MRP1/ABCC1 and MDR1/ABCB1 drug efflux pumps in A549 cells forming ALI multilayered mono-cultures grown for 14 d and then exposed to docetaxel (Doc), vinblastine (Vin), cytarabine (Cyt) or methotrexate (Met) at their nominal IC_{50} concentration for 72 h. Cell cultures were exposed to drugs by direct inoculation.

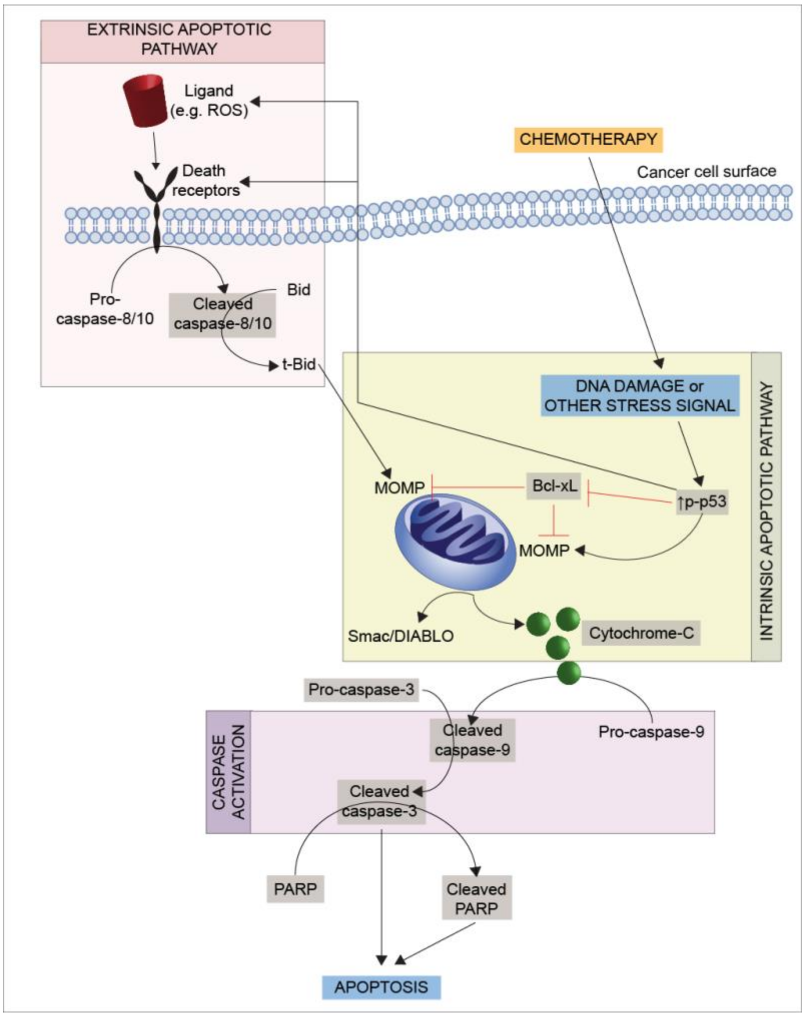

Figure S12. Schematics of the two apoptotic pathways (intrinsic and extrinsic) triggered by chemotherapy in cancer cells. The biomolecular markers analysed in this study are showed by the grey boxes. Abbreviations: MOMP, mitochondrial outer membrane permeabilization; PARP, poly(ADP-ribose) polymerase; ROS, reactive oxygen species.

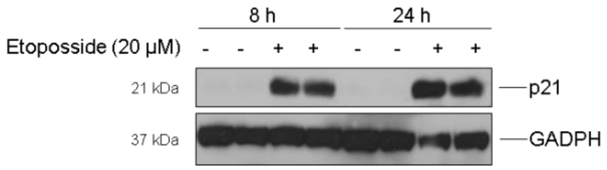

Figure S13. p21 expression in response to DNA damage. A549 cells were cultured as sub-confluent monolayer mono-cultures and treated with 20 μM etoposide for 8 and 24 h to induce DNA damage, or incubated with supplemented DMEM medium as negative control. Western blotting assay was used to examine expression of p21, a downstream target of p53. GADPH protein was also examined as a protein loading control. As for ATCC characterization[1], exposure to etoposide triggered p21 expression in A549 cells.

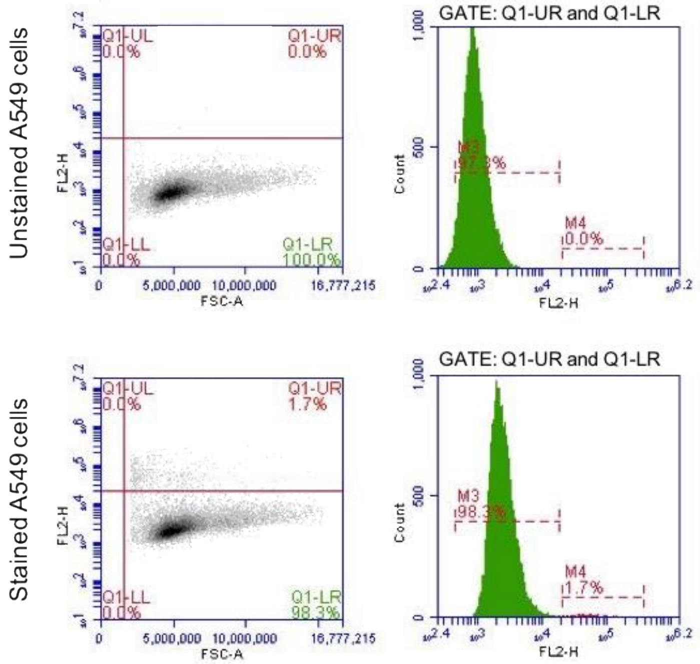

Figure S14. Representative gating applied for flow cytometry viability assay (LIVE/DEAD Fixable Red Dead Cell Stain Kit). In cells with compromised membranes, the dye reacts with free amines both in the cell interior and on the cell surface, yielding intense fluorescent staining (Q1-UR gate of scatter plots). In viable cells, the dye's reactivity is restricted to the cell-surface amines, resulting in less intense fluorescence (Q1-LR gate of scatter plots). Q1-LR gate was adjusted on the signal detected for unstained A549 cells (shown in the top row). Cell populations in Q1-UR and Q1-LR gates are also shown in histogram format, where the red fluorescence intensity (FL2-H) is plotted against the counts: M3 and M4 gates highlight the percentage of live and dead cells in the sample analysed, respectively.

SUPPORTING VIDEOS

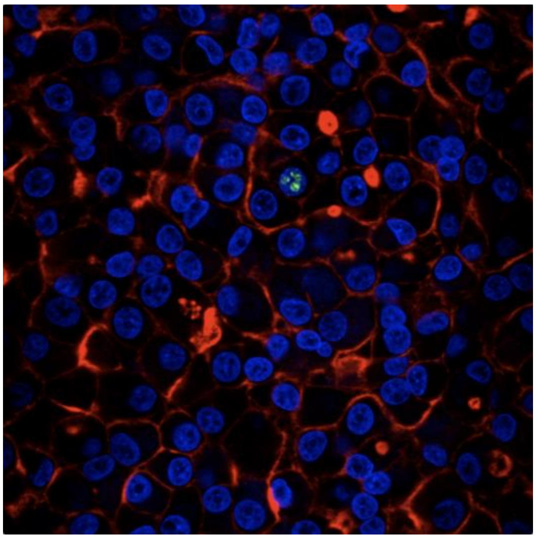

Video S1. Animated reconstruction of Z-stack LSCM images of ALI multilayered mono-cultures grown for 14 d and stained with: Hoechst 33342 for cell nuclei (in blue), rhodamine phalloidin for F-actin (in red) and Ki67 protein expression (in green). Objective lens: 63×.

SUPPORTING TABLES

Table S1. Co-localization of Ki67 protein expression and nuclear staining in ALI multilayered mono-cultures. MCCs were grown for various culturing time-points and then stained and imaged, as described in the material and methods section. The co-localization study was carried out on representative Z-planes of LSCM images by means of the ZEN software (ZEISS, Germany), setting an intensity threshold equal to 50 for both channels. An overlap coefficient equal to 1 indicates perfect co-localization of the Ki67 protein with cell nuclei, while no co-localization corresponds to an overlap coefficient of 0.

**Time-point**	**Overlap coefficient**
	**ALI multilayered mono-cultures**
24 h	0.70
48 h	0.79
72 h	0.59
7 d	0.80
14 d	0.86

Table S2. Statistical analysis (two-way ANOVA and Bonferroni post-test) on the differences in percentage (%) of live A549 cells and % cytotoxicity found in MCCs cultured for 14 d either on Transwell™ supports (in submerged conditions) or on plastic substrates (in submerged conditions) and then exposed to four anti-cancer drugs at their nominal IC_{50} concentration for 72 h, as compared to the responses of ALI multilayered mono-cultures.

**Parameter**	**Treatment**	**Multilayered mono-cultures**	
		**Submerged on Transwell™ support**	**Submerged on plastic substrate**
Live A549 cells (%)	Docetaxel	*ns*	$p<0.001$
	Vinblastine	$p<0.05$	$p<0.01$
	Cytarabine	$p<0.001$	*ns*
	Methotrexate	$p<0.001$	$p<0.01$
Cytotoxicity (%)	Docetaxel	*ns*	*ns*
	Vinblastine	$p<0.001$	$p<0.001$
	Cytarabine	$p<0.001$	$p<0.001$
	Methotrexate	$p<0.001$	$p<0.001$

SUPPORTING REFERENCES

1 ATCC. https://www.atcc.org/Documents/Learning_Center/~/media/5F7B1CCACF724E3398BE56BFBEE3EFE4.ashx *[Last access: August 2017]*.
